# A systematic review of antimicrobial resistance in *Salmonella enterica* serovar Typhi, the etiological agent of typhoid

**DOI:** 10.1371/journal.pntd.0006779

**Published:** 2018-10-11

**Authors:** Carl D. Britto, Vanessa K. Wong, Gordan Dougan, Andrew J. Pollard

**Affiliations:** 1 Oxford Vaccine Group, Department of Paediatrics, University of Oxford and the NIHR Oxford Biomedical Research Centre, Oxford, United Kingdom; 2 Wellcome Trust Sanger Institute and the Department of Medicine, Cambridge University, Cambridge, United Kingdom; Christian Medical College, INDIA

## Abstract

**Background:**

The temporal and spatial change in trends of antimicrobial resistance (AMR) in typhoid have not been systematically studied, and such information will be critical for defining intervention, as well as planning sustainable prevention strategies.

**Methodology and findings:**

To identify the phenotypic trends in AMR, 13,833 individual *S*. Typhi isolates, reported from 1973 to 2018 in 62 publications, were analysed to determine the AMR preponderance over time. Separate analyses of molecular resistance determinants present in over 4,000 isolates reported in 61 publications were also conducted. Multi-drug resistant (MDR) typhoid is in decline in Asia in a setting of high fluoroquinolone resistance while it is on the increase in Africa. Mutations in QRDRs in *gyrA* (S83F, D87N) and *parC* (S80I) are the most common mechanisms responsible for fluoroquinolone resistance. Cephalosporin resistant *S*. Typhi, dubbed extensively drug-resistant (XDR) is a real threat and underscores the urgency in deploying the Vi-conjugate vaccines.

**Conclusion:**

From these observations, it appears that AMR in *S*. Typhi will continue to emerge leading to treatment failure, changes in antimicrobial policy and further resistance developing in *S*. Typhi isolates and other Gram-negative bacteria in endemic regions. The deployment of typhoid conjugate vaccines to control the disease in endemic regions may be the best defence.

## Author summary

Typhoid is an invasive bacterial disease causing 26 million illness episodes globally, each year particularly in South Asia and sub-Saharan Africa afflicting children and poorer sections of society disproportionally. AMR is increasingly recognized among *S*. Typhi lineages spreading from South Asia to Africa, with resistance to first line antibiotics (co-trimoxazole, ampicillin and chloramphenicol), and fluoroquinolones and, of concern, cephalosporins which contribute to treatment failure. AMR in typhoid is not uniform globally and has evolved at different rates in various endemic regions. These trends have not been systematically analysed previously and the objectives of this study included reviewing the phenotypic and genetic determinants of AMR globally over time. The significance of this study revolves around identifying the different trends and mechanisms of AMR and planning interventional strategies accordingly, particularly in light of the Vi-conjugate vaccine candidate which recently received SAGE recommendation and WHO pre-qualification.

## Introduction

Enteric fever is a systemic infection, caused by the Gram-negative bacteria *Salmonella enterica* subspecies *enterica* serovars Typhi and Paratyphi A, that continues to be a significant cause of morbidity and mortality in endemic regions. Annually, it is estimated that over 26 million people are culture positive for *S*. Typhi/ Paratyphi[1], and a significant proportion of isolates are resistant to multiple antimicrobials[2]. South and South-East Asia, continue to be critical hubs for enteric fever, dominated by the H58 haplotype of *S*. Typhi in many regions. Fluoroquinolone resistance is widely prevalent across Asia, in part because of the widespread use of this class of antimicrobials.

Terminology to describe AMR in typhoid can be confusing, with the term MDR *S*. Typhi, historically used to describe combined resistance to chloramphenicol, co-trimoxazole (trimethoprim-sulfamethoxazole) and ampicillin. These antibiotics are frequently termed first-line antimicrobials in the literature as these were amongst the first to be recommended for typhoid treatment by the WHO[3]. MDR *S*. Typhi is now generally on the decline in South and South-East Asia, potentially because these drugs are no longer in common use, in view of the previous widespread resistance to these agents[4–7]. Empiric antimicrobial use for treating suspected typhoid fever in this region is now predominantly with third-generation cephalosporins including ceftriaxone and cefixime or azithromycin, since fluoroquinolone resistance is so common.

In contrast to the situation in Asia, MDR typhoid appears to be on the increase in parts of Africa. Several regions have reported typhoid outbreaks in the last decade and these have been associated with MDR phenotypes. H58 *S*. Typhi disease is moving through areas of East and Southern Africa, while, non-H58 haplotypes are implicated in the Western and Northern regions, illustrating the heterogeneous nature of the disease on the continent[8,9].

The historical trend of antibiotic sensitivity and resistance in *S*. Typhi has not been systematically analysed and reported. Understanding this trend is important and may provide clues for sustaining treatment regimens in endemic areas as well as modelling the potential impact of typhoid vaccines in reducing AMR. This study uses a global genotypic and phenotypic approach to summarise such trends.

## Methods

**Fig 1:**
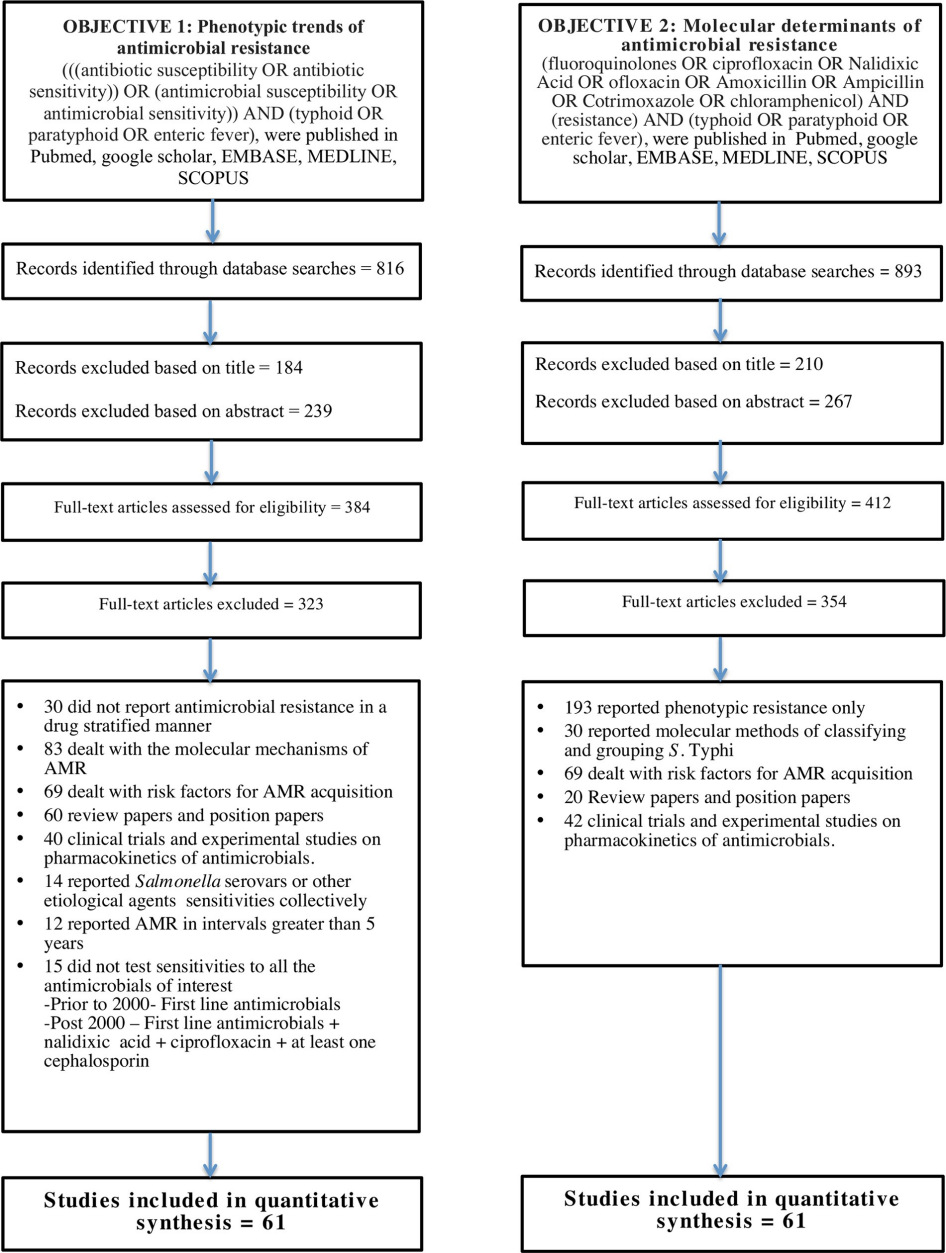
Search strategy and PRISMA flow-diagram.

The objectives of this review were two-fold: to systematically delineate the historical trend of expressed phenotypic resistance to first-line antimicrobials, nalidixic acid, ciprofloxacin and cephalosporins as well as to describe the molecular mechanisms of AMR in typhoid. The search strategies for both objectives are described in **Fig 1**. Exclusion criteria such as time of publication, study design and language were not applied in the search builder in order to ensure complete data collection.

### Phenotypic trends in antimicrobial resistance

An isolate was considered resistant to an antimicrobial if it was reported as “resistant”, “intermediately susceptible”, “intermediately resistant” or “non-susceptible” based on minimum inhibitory concentration (MIC) values or diameters of zones of inhibition *via* disc diffusion using customary interpretive criteria such as the Clinical & Laboratory Standards Institute (CLSI) or the European Committee on Antimicrobial Susceptibility Testing (EUCAST) standards. For consistency, studies prior to the year 2000, that reported sensitivities of at least the first-line antimicrobials were included while studies conducted after the year 2000, which did not report antimicrobial sensitivities of either chloramphenicol, co-trimoxazole, ampicillin/amoxicillin, nalidixic acid, ciprofloxacin or at least one cephalosporin were excluded. Studies that reported antibiograms collectively and had not stratified these into intervals shorter than 5 years were also excluded.

Isolates identified from reports were then stratified based on year of isolation, geographic location and resistance phenotypes. Stratified isolates that were resistant to each antimicrobial were then expressed as a proportion of all the isolates reported. The trends of antimicrobial resistance were then expressed in 5-year intervals as represented in **Table 1**. This process was then repeated on isolates collected from Asia and Africa separately.

**Table 1:** Proportion of antimicrobial non-susceptibility stratified in 5 year intervals.

Year	Total no. of isolates	Proportion (%) of resistant isolates						
		CH	AMP	TMX	MDR	NAL	CIP	CEPH
Pre-1991	507	31.2	16.2	16.1	**16.1**	NA	NA	NA
1991–1995	2506	49.2	49.1	49.2	**49.1**	NA	NA	NA
1996–2000	2436	44.1	46	45	**44**	22	12	2
2001–2005	4654	31	35	33	**31**	50	23	1
2006–2010	1974	19	32	18	**18**	63	33	1
2011–2015	1756	13	20	18	**13**	8	63	4

Table 1 represents the proportions of antimicrobial non-susceptibility stratified in 5- year intervals. These data were pooled from 72 published reports from 1973 to 2017

Abbreviations; CH-Chloramphenicol, AMP-Ampicillin, TMX-Cotrimoxazole, NAL-Nalidixic Acid, CIP-Ciprofloxacin, CEPH-Cephalosporins

### Molecular determinants of antimicrobial resistance

For the second objective, studies reporting molecular mechanisms of AMR of isolates either collectively or individually were included. These were only stratified based on country of isolation and type of mechanism reported as methods used to study these mechanisms were heterogeneous over the years and techniques employed have also changed drastically thus making temporal comparisons challenging.

### Data extraction

Data from individual studies were extracted under the following parameters: (i) **study identifier**: first author, year of publication, year of study commencement, duration of study, country, study design and sampling population (hospital-based/ community and travel-associated/endemic or outbreak); (ii) **methodology**: sample size, site of isolation and antimicrobial susceptibility testing, interpretive criteria. For the studies included to study molecular determinants the technique of molecular detection was also recorded. (iii) **results:** numbers of *S*. Typhi isolates, frequency of MDR, nalidixic acid resistant, fluoroquinolone resistant and cephalosporin resistant strains. In addition we also collected data o the molecular mechanisms of MDR, fluoroquinolone and cephalosporin resistance in the form of AMR determining genes, resistance plasmids and AMR conferring SNPs. Study-specific data extraction was done twice–overall all for objective 1 and objective 2 separately.

### Risk of bias

Inclusion criteria were used to establish study validity. Risk of bias (RoB) was assessed using two tools (**S3 Table**). The first classifies studies based low-, moderate- or high- risk of bias and is known as the Quality In Prognosis Studies tool (QUIPS)[10]. The second is known as the Joanna Briggs Institute (JBI) tool[11] and reports RoB dichotomously. The JBI was adapted for use in this study similar to the adaptations used by Tadesse *et al* [12] We performed these RoB analysis separately on studies selected to meet the first and second objective. The isolates derived from these studies were then used for the frequency analysis. Parameters assessed for bias across the two tools included 1) Population description, i.e. whether community or hospital setting, 2) Study design, sample size and sampling techniques 3) Use of appropriate performance standards and quality control in microbiologic techniques such as bacteriologic culture and antimicrobial sensitivity and 4) the statistical analysis used for reporting summary measures.

## Results

### Phenotypic trends of antimicrobial resistance

In order to estimate frequencies of antimicrobial resistance in *S*. Typhi we set key criteria for such an analysis (See Methods). We initially focused on phenotypic data collected through classical antimicrobial susceptibility testing. Here, sixty-two studies **(S1 Table)** satisfied the inclusion criteria from which a cumulative number of 148 year-stratified summaries of antimicrobial resistant *S*. Typhi isolates were obtained. For example, Rahman *et al*[13] reported the isolates of their study in a year-stratified manner for 13 years, therefore providing 13 serial year-stratified summaries. Of our accepted 148 year-stratified summaries, 37 were undertaken prior to the year 2000 and more than 80% were retrospective in study design. The year-stratified summaries obtained from each report were then pooled into the following temporal intervals; pre-1991, 1991–1995, 1996–2000, 2001–2005, 2006–2010 and 2011–2015 and expressed as a proportion of resistant isolates for each antimicrobial **(Table 1)**. In addition to RoB estimation for each included study that suggested most studies were in the spectrum of medium to low risk of bias, the confidence intervals estimated for each year-stratified summary further suggested that majority of data points in each temporal period contributed significantly to the overall trends and is illustrated in **S1 Fig**.

Of the 13,833 isolates obtained from the various reports, 63.2% were isolated from South Asia, 12.8% were from South-East Asia, 15% were from the continent of Africa mostly represented by countries in the East and South-West regions. The spatial distribution of isolates from endemic settings is illustrated in S2 Fig. Isolates that were cultured from travellers returning from endemic regions made up the remainder of the isolates included in this analysis. The number of isolates within each time interval rose steadily until 2001–2005, a period that accounted for the most isolates (4,725 isolates), the subsequent time intervals saw a decline in published data.

**Fig 2:**
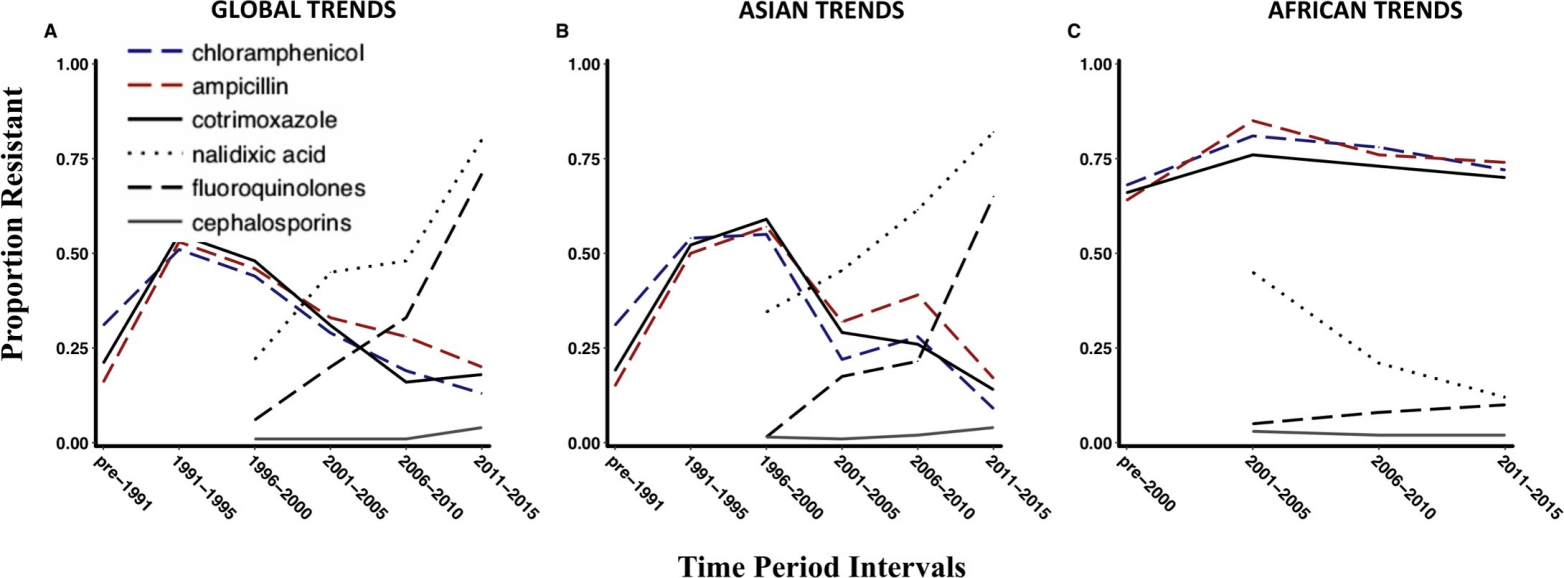
Antimicrobial non-susceptible trends of *S*. Typhi over time A) Global trends, B) Trends in Asia C) Trends in Africa. Fig 2A is Graphical representation of the proportion of S. Typhi isolates obtained from reports that were resistant to antimicrobials (indicated by coloured lines). Isolates represented in this graph were consolidated from published reports between 1973 and 2017 from endemic and epidemic sources, assembled systematically. In comparison to Fig 2A, Fig 2B represents the AMR trends obtained from Asian reports. Note the similarity in the trend between 2a and 2b; it is evident that non-susceptibility to first-line antimicrobials (chloramphenicol, co-trimoxazole and ampicillin) has decreased over time. Fig 2C represents the AMR trends from African reports. MDR Typhoid is widely prevalent while fluoroquinolone resistance is low.

Nalidixic acid, ciprofloxacin and cephalosporin trends were only analysed from the late 1990’s as these drugs were not routinely tested as part of antimicrobial sensitivity studies prior to this period, although preliminary reports of ciprofloxacin resistance surfaced as early as 1992[14]. **Fig 2A** summarises the global AMR trends, which indicate a trend of decline in MDR and an increasing level of fluoroquinolone resistance.

The temporal distribution of isolates obtained from Asia and Africa, when analysed independently, revealed very different trends as shown in **Fig 2B and 2C** respectively. The proportion of MDR *S*. Typhi in Asia saw declining trends, accounting for less than 20% of isolates obtained between 2011 and 2015, whereas resistance to nalidixic acid and fluoroquinolones continued to increase during this period (from 20% in 2001–2005 to 65% in 2011–2015), prompting a change to the use of third-generation cephalosporins in the treatment of enteric fever. Third-generation cephalosporin resistance rose from 1.5% in the 2006–2010 to 4% in the 2011–2015 time interval. Azithromycin is now often used for the treatment of enteric fever, but the number of reports on the susceptibility did not meet the inclusion criteria for this systematic review and were too few to be presented in this study. However, there are sporadic reports of phenotypic resistance[15–17].

In Africa the scenario is very different, where MDR typhoid is still common, with over 90% resistance in some regions. Interestingly, fluoroquinolone and third-generation cephalosporin resistance are still low (< 1%).

### Molecular determinants of antimicrobial resistance

To meet the second objective of this review 4,226 isolates spanning 61 studies (**S2 Table**) were included for the analysis of molecular mechanisms. Most studies (66%) incorporated the polymerase chain reaction (PCR) method to study the molecular determinants of antimicrobial resistance. However, four studies[4,8,9,18] reported whole genome sequence analysis of 2,118 isolates and between them provided valuable insights into the development of resistance in *S*. Typhi at a molecular level. In keeping with the phenotypic trends of AMR, the molecular findings of isolates between Africa and Asia were contrasting.

**Fig 3:**
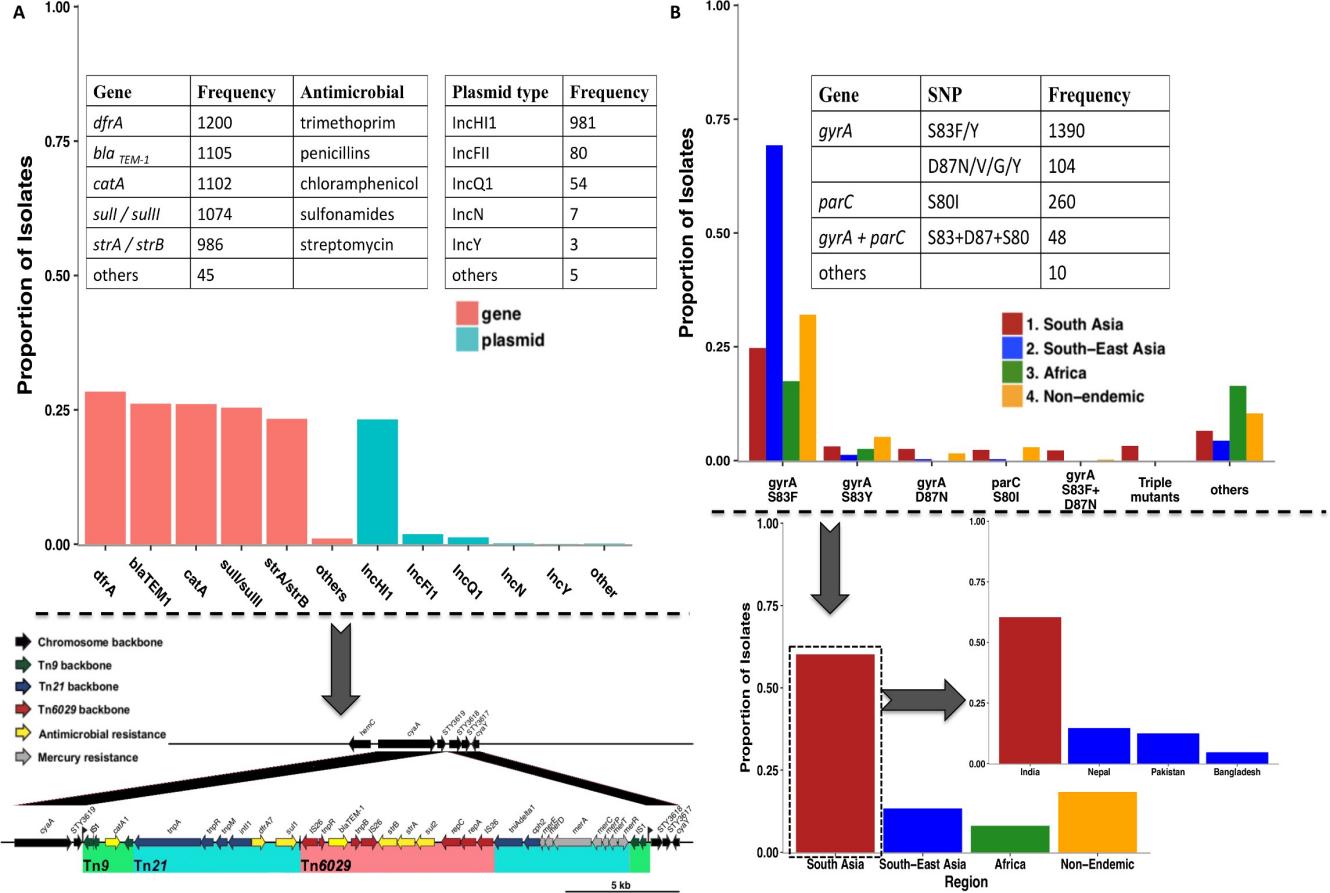
Summary of the molecular determinants of resistance in *S*. Typhi as identified in this review. Panel A is a graphical representation of MDR genes and plasmids with the frequency of each inset as a matrix indicating the resistance gene and corresponding antimicrobial. The arrow indicates the arrangement of MDR genes in the plasmid or the location within the bacterial genome. Panel B illustrates the proportion of isolates harbouring fluoroquinolone proportion of isolates derived from the 4 main South Asia countries. resistance determining SNPs and the regions of origin of isolates, the vertical arrow indicates to the graph illustrating the proportion of isolates retrieved from each region and the horizontal arrow points to the graph depicting the proportion of isolates from each of the South Asian countries. Fluoroquinolone resistance occurs through mutations DNA gyrase enzyme of the bacteria which is encoded by *gyrA*, *parC* and *parE* as mentioned in the matrix inset.—SNP–Single Nucleotide Polymorphism- Amino acid abbreviations S–Serine, F- Phenylalanine, Y–Tyrosine, D–Aspargine, N–Aspartic acid, I–Isoleucine.

Genetic signatures associated with fluoroquinolone resistance were very distinct amongst isolates studied in Asia (**Fig 3B**). Single nucleotide polymorphisms (SNPs) in *gyrA*, *gyrB*, *parC* and *parE*, which include the quinolone resistance determining region (QRDR) in the *S*. Typhi genome, as well as fluoroquinolone resistance conferring plasmids containing *qnrB2*, *qnrB4* and *qnrS1* genes were reported. From these data it is apparent that fluoroquinolone resistance in *S*. Typhi is frequently linked to mutations with *gyrA*. A frequent position for SNPs in *gyrA* is codon 83 with the S83F being most common occurring in 1189 isolates. S80I was the most common SNP in the *parC* gene, detected in 260 isolates, together with a concordant SNP in S83F. The S83Y mutation was detected in 209 isolates, while 57 isolates harboured the mutation *gyrA* D87N, further underpinning the importance of *gyrA*–associated SNPs, likely in response to antimicrobial selection pressure. Isolates harbouring combinations of three SNPs in *gyrA*, at codons 83 and 87 as well as mutations at codon 80 in *parC* are associated with a high level of ciprofloxacin resistance and designated as ‘triple mutants’. These triple mutations were mostly commonly identified in *S*. Typhi isolates from South Asia[4,9], often in distinct sub-groups within the main H58 clonal population[4]. SNPs in *parE* and *gyrB* were also observed but to a much lower extent (3 and 7 isolates respectively). The *qnrB2*, *qnrB4* and *qnrS1* resistance determinants have been found in *S*. Typhi but they are still rare, being identified in 21 *S*. Typhi isolates from Asia. These are usually encoded on plasmids. We can anticipate that such isolates may become more common in the future.

The relatively recent trend to a decline in MDR *S*. Typhi in Asia has been accompanied by a decrease in the proportion of isolates carrying IncHI1 plasmids, which often harbour the resistance genes responsible for MDR typhoid. Such resistance genes are clustered on composite transposons and include *catA*, *sul1*, *sul2*, *dfrA*, *bla*_*TEM-1*_, *strA*, *strB*, *tetA*, *tetB*, *tetC and tetD*. These MDR-associated genes can also be found integrated on the chromosome of H58 *S*. Typhi in isolates from countries including India and Bangladesh[9]. The arrangement of these genes and transposons both in plasmids and embedded in the bacterial chromosome are illustrated in **Fig 3A**. Other plasmids identified in *S*. Typhi included R27-like, B7-like and those falling into IncH and IncN, but these are currently relatively uncommon.

The scenario in Africa was very different with MDR being widely prevalent, conferred in part by determinants encoded on IncHI1 plasmids. The H58 clade of *S*. Typhi is associated with much of the typhoid occurring in the last decade in East and Southern Africa, although other haplotypes do occur. The situation is somewhat different in Western Africa, where H58 is still uncommon and AMR typhoid is spread via non-H58 clades[8] with both IncH1 and IncY plasmids being present in the circulating population. Again, as elsewhere in Africa, genetic signatures of fluoroquinolone resistance were present in only a few of the analysed isolates. SNPs in *gyrA*, *gyrB*, *parC* and *parE* were detected in 36 isolates with the S83F SNP in *gyrA* being the most common. Plasmids encoding the *qnrB2*, *qnrB4* and *qnrS1* determinants have also been reported. Other plasmid-types identified in Africa are illustrated in **Fig 3A.**

Extended spectrum β lactamase (ESBL) producing *S*. Typhi isolates, which confer resistance to third-generation cephalosporins have been reported in India and Pakistan[19,20]. The Indian isolates carried IncX3 and IncA plasmids which encoded *bla*_SHV-12_ and *bla*_CMY-2_ determinants[19], as well as *bla*_TEM-1B_ and *bla*_DHA-1_ probably on an IncN plasmid[21]. More recently, a case report of *S*. Typhi encoding ESBL (*bla*_*C*TX-M15_) on a IncY type plasmid has been reported in the Democratic Republic of Congo[22]. Other CTX-M producing isolates have been reported from Southern India[23], Nigeria[24], Japan[25] as well as from travellers returning from Guatemala[26] and Iraq[27]. A recent publication reported *bla*_CTX-M15_ producing *S*. Typhi isolates from Pakistan that were cephalosporin resistant in addition to MDR and fluoroquinolone resistant and have been labelled as XDR (extensively drug resistant). All the XDR isolates had a composite transposon as described above and an additional IncY plasmid containing *bla*_CTX-M15_ and *qnrS* genes[18].

There were also reports of azithromycin resistance mediated *via* the *ereA* from an isolate from Algeria, as well as *via msrD* and *msrA* from an Indonesian isolate[9].

## Discussion

The paucity of reliable point of care diagnostics for typhoid fever compels clinicians in the field to initiate presumptive antimicrobial therapy, often based on clinical judgment. In endemic settings, typhoid features high on the list of potential causes of undifferentiated febrile illness, and antimicrobial therapy is routinely started empirically with antimicrobials that are thought to be appropriate for local clades of *S*. Typhi. The data presented in this systematic review suggest that such antimicrobial use for the treatment of presumptive enteric fever is likely influencing the patterns of AMR in *S*. Typhi.

Two independent published reports entail global antimicrobial consumption trends. The first report assessed antimicrobial consumption between 2000 and 2010 and suggested that global antibiotic consumption increased by 36% based on national pharmaceutical sales. Most notably in 2010, India and China were the world’s first and second largest consumers of antibiotics respectively[28]. In India and similarly in other LMIC settings, the three classes of antimicrobials that were most consumed were beta-lactams, macrolides, and fluoroquinolones. The second report published in 2018 gauged antimicrobial consumption between 2000 and 2015 in defined daily doses (DDDs) as well in DDDs/1000 inhabitants/day and suggested that antimicrobial consumption has increased by 65% between 2000 and 2015 globally and interestingly showed that India and Pakistan were the two of the top three largest antimicrobial consumers. This rise was attributed to the to changing prescribing practices favouring cephalosporins for enteric fever among other virulent infections involving the respiratory tract, genito-urinary tract, skin and soft tissue in the setting of rising AMR for other antimicrobials including narrow spectrum beta-lactams and fluoroquinolones[29].

For the treatment of enteric fever, the first-line antimicrobials (chloramphenicol, co-trimoxazole and ampicillin) were recommended between 1948 and the early 1990s.[14] Unfortunately, the widespread use of these drugs facilitated the emergence of resistance to chloramphenicol and subsequently to ampicillin and co-trimoxazole, leading to MDR typhoid[14]. MDR typhoid became established in parts of Asia in the 1990’s and the phenotype was mainly conferred through the acquisition of horizontally acquired plasmids[14] harbouring transposons and integrons encoding resistance-determining genes. The most commonly implicated plasmids found in *S*. Typhi at this time were of the IncHI1 type[14,30,31]. Bayesian analysis suggests that this plasmid was first acquired by H58 and some other haplotypes of *S*. Typhi in Asia around the early 1990s[9]. With the establishment of widespread MDR typhoid, the use of chloramphenicol, ampicillin and co-trimoxazole became obsolete in this region. However, this analysis indicates that the subsequent circulation of these plasmids within *S*. Typhi in Asia markedly decreased over time, highlighting the adaptability of *S*. Typhi to changing antibiotic pressure.[4,32] The Global AMR trends in **Fig 2** are driven very strongly by the trends in Asia and this is likely to be related to the magnitude of burden in South and South-East Asian countries, the relatively recent endemicity of the disease in Africa as well as a potential reporting bias related to under-reporting by African regions. Although this review did not attempt to estimate the true burden of antimicrobial resistant typhoid the contribution of Asian isolates to the over-all global trends is conspicuous in **Table 1**, **Figs 2 and 3**. Further, **Fig 3B** illustrates the striking contribution of isolates from Indian studies in determining Asian trends. The highest proportion of isolates analysed in this study came from India (30.4%), Bangladesh (30.1%) and Vietnam (14.4%). Among the African countries Nigeria (2.6%), Kenya (0.7%) and Ghana (0.54%) accounted for the largest proportion of total study isolates. The spatial distribution of isolates is depicted in **S2 Fig**. The trend observed in Africa is very different and may partially reflect the more recent introduction of *S*. Typhi isolates into the continent[9]. Transposon-mediated MDR typhoid associated with composite transposons either on plasmids or in the chromosome is increasingly reported, driven by both H58 and non-H58 clades[8]. Although IncHI1 plasmids are still the most commonly identified, other incompatibility (defined as the inability of two related plasmids to be stably transmitted together[33]) group plasmids such as IncY, IncN and IncFIIK (pKPN3) have also been identified in *S*. Typhi in Africa[8].

Following the emergence of MDR typhoid, fluoroquinolones were adopted as the treatment of choice for typhoid by the late 1990’s. The fluoroquinolone class of antimicrobials were highly effective, could be orally administered, had minimal side effects and had rapid rates of bacteraemic clearance times although potential adverse effects on the growing epiphysis of long bones was viewed with suspicion and initially restricted in children[14]. Nevertheless, ciprofloxacin and ofloxacin became favoured alternatives to the former first-line antimicrobials and consequently fluoroquinolone resistance began to develop. The antimicrobial pressure associated with fluoroquinolone usage likely facilitated the acquisition of alternative modes of antimicrobial evasion by *S*. Typhi. The spread of fluoroquinolone resistance was accelerated by the emergence of the H58 clade, which dominated circulating *S*. Typhi populations by the late 1990s, with an apparent increased fitness advantage and enhanced transmission success[9,34]. Unlike resistance to first-line antimicrobials, resistance to fluoroquinolones was mediated *via* the accumulation of non-synonymous SNPs in the genome inducing conformational changes in DNA gyrase and topoisomerase IV, the main sites of fluoroquinolone action. The genes in which SNPs occur include *gyrA*, *parC*, *parE* and *gyrB*, with *gyrA* SNPs correlating strongly with treatment failure[4]. Unfortunately, the standard method of gauging antimicrobial sensitivity, i.e. disc diffusion, suggested that *S*. Typhi was still relatively sensitive to ciprofloxacin despite ongoing treatment failure and relapse. A WHO report comprising of multi-centric antimicrobial surveillance data of typhoid isolates across India between 2008–2010 suggested that nalidixic acid sensitivity was a good indicator of fluoroquinolone sensitivity but there was a disparate correlation with nalidixic acid resistance and ciprofloxacin resistance[35] which is exemplified by the dissimilar nalidixic acid and ciprofloxacin trend lines in **Fig 2A and 2B**. These observations concluded that nalidixic acid break points on disc diffusion correlated more accurately with ciprofloxacin sensitivity, prompting a revision in the CLSI recommended break points in 2012[36]. An Indian study compared breakpoints for ciprofloxacin using the CLSI guidelines before and after the 2012 revision and also with the EUCAST guidelines and found that only 3% of isolates were sensitive using the revised guidelines vs 95% of isolates that were sensitive using the older guidelines[36]. The sensitivities of isolates reported using EUCAST breakpoints were comparable to the revised CLSI breakpoints[36]. The nalidixic acid and ciprofloxacin trend lines in **Fig 2A and 2B** which seem to converge may in reality be attributed to revisions in the CLSI guidelines for ciprofloxacin breakpoints. Fluoroquinolone-resistant *S*. Typhi isolates are currently widespread in Asia with over 60% of isolates in this review demonstrating resistance. In **Fig 3B** the proportion of isolates harbouring fluoroquinolone resistance conferring SNPs from South-Asia appears to be less that the proportions of South-East Asia and this is mainly due to two factors; the total number of isolates obtained during data extraction were dominated by reports from South Asia but a substantial proportion of these isolates were obtained prior to the era of widespread fluoroquinolone resistance, secondly it is difficult to do any temporal analysis as these results will be subject to high rates of bias due to the different methods employed in studying genetic determinants which have varied over time. For instance, the PCR, pulse field gel electrophoresis and conjugation transfer to *E*. *Coli* techniques employed do not always look for all MDR, fluoroquinolone and cephalosporin determinants of resistance, where as this is possible with whole genome sequencing resulting in broader information of AMR determinants. In Africa, 90% of isolates are still susceptible to fluoroquinolones with some reports of *gyrA* SNPs recently emerging[37][12]. Accumulating mutations in the QRDR cause *S*. Typhi to gradually increase the MIC values of ciprofloxacin. Ciprofloxacin-susceptible strains (MIC—0.06 μg ml) are known to acquire a *gyrA* S83F single mutation with a subsequent increase in MIC values (0.12–0.5 μg ml) and additional *gyrA* and *parC* mutations continue to cause an increase in MICs up to 4 μg ml[38].

More recently, third-generation cephalosporins and azithromycin have become the preferred treatment choices for typhoid in the face of MDR and fluoroquinolone resistance, owing to the broad spectrum of activity and the option of oral or intravenous administration. Nevertheless, widespread third-generation cephalosporin resistant typhoid is now on the horizon in South Asia with sporadic reports of treatment failure from India[19,21] and Pakistan[18,20,39] and the XDR typhoid outbreak in populous parts of the Sindh province in Pakistan[18]. In South Asia, cephalosporins such as ceftriaxone and cefixime are currently the mainstay of treatment for enteric fever, and are often started empirically, likely driving resistance in typhoid and other Gram-negative bacteria.

Confirmed typhoid and paratyphoid infections make up only a minority of the total proportion of all Gram-negative infections in endemic regions[40,41]. However, empirical antimicrobial treatment with cephalosporins for presumptive enteric fever confers an antimicrobial pressure, which encompasses all Gram-negative bacterial populations. It is thus plausible that the impact of empiric therapy for typhoid is of far greater importance in driving AMR than just as described in this study in *S*. Typhi. The mechanisms of resistance adopted by *S*. Typhi are similar to those among other Gram-negative bacteria [42] and the most contemporary concern stems from the emergence of extended spectrum β lactamases (ESBLs) produced by various Gram-negative species, which has originated as a result of the widespread cephalosporin use. Preventive approaches warrant a collective approach in tackling Gram-negative resistance as the molecular determinants of resistance are transferrable between Gram-negative organisms and thus reducing the use of cephalosporins for typhoid is likely to have an indirect effect on the other Gram-negative organisms [42].

A 2014 publication suggested that cephalosporins were the most commonly used antimicrobial in India and China, followed by broad-spectrum penicillins, fluoroquinolones and macrolides[28]. This trend might still hold true in 2017 which highlights the mounting antimicrobial pressure exerted by the use of cephalosporins culminating in the production of ESBLs by Gram-negatives, including *S*. Typhi.[19–21] These issues underscore the importance of controlling the spread of typhoid through the deployment of vaccines and prudent antimicrobial use in the short-term.

Single drug therapy (monotherapy) has been common practice in the treatment of typhoid, and monotherapy with former first-line antimicrobials may be a reasonable option in Asia. A single report from Nepal suggests that monotherapy with co-trimoxazole results in complete remission of typhoid fever caused by H58 which was fluoroquinolone-resistant but not MDR[43]. However, a more astute approach in Asia might involve combination therapy with a first-line antimicrobial and perhaps azithromycin. This approach for the treatment of enteric fever in Asia could potentially facilitate the conservation of cephalosporins. The decrease in MDR highlighted in this review following the reduction in use of first-line antibiotics (amoxicillin, chloramphenicol and co-trimoxazole) shows that cycling of these antibiotics for control of typhoid might be an option, where close monitoring of susceptibility is feasible. However, uncoordinated use of these agents would likely lead to a rapid re-emergence of MDR and it is difficult to see how such a programme could be undertaken globally. Immunization could theoretically reduce the number of circulating MDR, fluoroquinolone- and cephalosporin-resistant strains and, furthermore, decrease the incidence of undifferentiated febrile illness thereby reducing the need for empirical antimicrobial therapy.

This study has limitations in that the interpretive criteria employed by majority of studies was the CLSI guidelines which was improved periodically particularly with regard to ciprofloxacin breakpoints in 2012. It is hard to ascertain how quickly individual laboratories made the transition after each revision. Finally, it is also unlikely that true trends of Asian and African isolates are not represented in its entirety, which is mainly due to the lack of published data. Regions from West and Central Africa as well as regions from South-East Asia were under-represented. It was also difficult to account for methodological variations in studying molecular determinants of AMR over time with the rapid evolution of molecular techniques.

*S*. Typhi rapidly acquires resistance to the antimicrobials that are being used in the community, but can also lose resistance once these drugs are withdrawn. From these observations, it seems likely that antimicrobial resistance will emerge in areas endemic for typhoid, leading to treatment failure, changes in antimicrobial policy and further resistance developing in *S*. Typhi isolates and other Gram negative bacteria. Therefore, deployment of typhoid conjugate vaccines to control the disease may be the best defence against antimicrobial resistance in *S*. Typhi.

## Supporting information

S1 Table Characteristics of publications included in the phenotypic analysis of AMR. (DOCX)

S2 Table Characteristics included publications included in the molecular analysis of AMR. (DOCX)

S3 Table Risk of bias assessment. (DOCX)

S4 Table PRISMA checklist. (DOC)

S5 Table Year-stratified summaries from studies included in this review. (XLSX)

S1 Fig Confidence intervals of the year stratified summaries in each temporal period. Those including 0 do not contribute significantly to the overall trends in Fig 2 and did not change the overall trends on sensitivity analyses.
(DOCX)

S2 Fig The spatial distribution of isolates from endemic areas analysed systematically in this review. (JPG)
