# Supplementary material for: A systematic review of antimicrobial resistance in Salmonella enterica serovar Typhi, the etiological agent of typhoid
Source: PLoS Negl Trop Dis. 2018 Oct 11;12(10):e0006779. doi: 10.1371/journal.pntd.0006779 (PMC6198998; doi:10.1371/journal.pntd.0006779)
Supplement: S1 Table — (DOCX) [file pntd.0006779.s001.docx]

| **Supplementary Table 1: Characteristics of publications included in the phenotypic analysis of AMR** | | | | | | | |
| --- | --- | --- | --- | --- | --- | --- | --- |
| **No.** | **Year of**  **Study** | **Year of Publication** | **Author** | **Country/Region of study** | **PubMed**  **IDentifier** | **Number of isolates** | **Study Design** |
| 1 | 2014 | 2017 | Ali A | Pakistan | 28303985 | 155 | Retrospective |
| 2 | 2012 | 2017 | Harichandran D | India | 28352198 | 79 | Retrospective |
| 3 | 2016 | 2016 | Osbourne LG | Travel associated | 26243802 | 1 | Case Report |
| 4 | 2016 | 2013 | [Sharvani R](https://www.ncbi.nlm.nih.gov/pubmed/?term=Sharvani%20R%5BAuthor%5D&cauthor=true&cauthor_uid=27437211) | India | 27437211 | 167 | Retrospective |
| 5 | 2013-2014 | 2016 | Misra R | India | 25979527 | 50 | Retrospective |
| 6 | 2014 | 2015 | [Khanam F](https://www.ncbi.nlm.nih.gov/pubmed/?term=Khanam%20F%5BAuthor%5D&cauthor=true&cauthor_uid=25849611) | Bangladesh | 25849611 | 72 | Retrospective |
| 7 | 2013 | 2015 | [Mahende C](https://www.ncbi.nlm.nih.gov/pubmed/?term=Mahende%20C%5BAuthor%5D&cauthor=true&cauthor_uid=26138060) | Tanzania | 26138060 | 17 | Prospective |
| 8 | 2015 | 2015 | [Narain U](https://www.ncbi.nlm.nih.gov/pubmed/?term=Narain%20U%5BAuthor%5D&cauthor=true&cauthor_uid=26388636) | India | 26388636 | 220 | Prospective |
| 9 | 2002-2013 | 2015 | Nüesch-Inderbinen M | Travel associated | 25963025 | 192 | Retrospective |
| 10 | 2012 | 2014 | [Jessica Maltha](https://www.ncbi.nlm.nih.gov/pubmed/?term=Maltha%20J%5BAuthor%5D&cauthor=true&cauthor_uid=24551225) | Burkina Faso | 24551225 | 12 | Prospective |
| 11 | 2012 | 2014 | [Srirangaraj S](https://www.ncbi.nlm.nih.gov/pubmed/?term=Srirangaraj%20S%5BAuthor%5D&cauthor=true&cauthor_uid=24817913) | India | 24817913 | 16 | Retrospective |
| 12 | 2008 | 2014 | [Chiou CS](https://www.ncbi.nlm.nih.gov/pubmed/?term=Chiou%20CS%5BAuthor%5D&cauthor=true&cauthor_uid=25136011) | Bangladesh | 25136011 | 38 | Retrospective |
| 13 | 2012 | 2014 | [Chand HJ](https://www.ncbi.nlm.nih.gov/pubmed/?term=Chand%20HJ%5BAuthor%5D&cauthor=true&cauthor_uid=25390062) | Nepal | 25390062 | 56 | Prospective |
| 14 | 2010 | 2014 | [Isendahl J](https://www.ncbi.nlm.nih.gov/pubmed/?term=Isendahl%20J%5BAuthor%5D&cauthor=true&cauthor_uid=25526763) | Guinea-Bissau | 25526763 | 3 | Prospective |
| 15 | 2014 | 2014 | [Dahiya S](https://www.ncbi.nlm.nih.gov/pubmed/?term=Dahiya%20S%5BAuthor%5D&cauthor=true&cauthor_uid=28303820) | India | 28303820 | 380 | Retrospective |
| 16 | 2010 | 2013 | [Choudhary](https://www.ncbi.nlm.nih.gov/pubmed/?term=Choudhary%20A%5BAuthor%5D&cauthor=true&cauthor_uid=23703350) A | India | 23703350 | 322 | Retrospective |
| 17 | 2012 | 2013 | Sultan BA | Pakistan | 23905456 | 1 | Case Report |
| 18 | 2013 | 2013 | [Vlieghe E](https://www.ncbi.nlm.nih.gov/pubmed/?term=Vlieghe%20E%5BAuthor%5D&cauthor=true&cauthor_uid=24094060) | Cambodia | 24094060 | 31 | Retrospective |
| 19 | 2012 | 2013 | [Venkatesh BM](https://www.ncbi.nlm.nih.gov/pubmed/?term=Venkatesh%20BM%5BAuthor%5D&cauthor=true&cauthor_uid=24441263) | India | 24441263 | 251 | Retrospective |
| 20 | 2008-2010 | 2013 | Gupta V | India | 24043999 | 257 | Retrospective |
| 21 | 2010-2012 | 2013 | Jain S | India | 24240035 | 266 | Retrospective |
| 22 | 2012 | 2012 | [Lutterloh E](https://www.ncbi.nlm.nih.gov/pubmed/?term=Lutterloh%20E%5BAuthor%5D&cauthor=true&cauthor_uid=22357702) | Malawi-Mozambique border | 22357702 | 42 | Retrospective |
| 23 | 2012 | 2012 | [Olut AI](https://www.ncbi.nlm.nih.gov/pubmed/?term=Olut%20AI%5BAuthor%5D&cauthor=true&cauthor_uid=22399179) | Turkey | 22399179 | 1 | Case Report |
| 24 | 2010 | 2012 | [Acharya D](https://www.ncbi.nlm.nih.gov/pubmed/?term=Acharya%20D%5BAuthor%5D&cauthor=true&cauthor_uid=22627312) | Nepal | 22627312 | 114 | Retrospective |
| 25 | 2008 | 2011 | [Kumar Y](https://www.ncbi.nlm.nih.gov/pubmed/?term=Kumar%20Y%5BAuthor%5D&cauthor=true&cauthor_uid=21444993) | India | 21444993 | 128 | Retrospective |
| 26 | 2011 | 2011 | Adhikary | India | 22234135 | 2 | Case Report |
| 27 | 2001-2002 & 2009 | 2011 | Gross U | Ghana | 22000360 | 74 | Retrospective |
| 28 | 2007 | 2010 | [Nagshetty K](https://www.ncbi.nlm.nih.gov/pubmed/?term=Nagshetty%20K%5BAuthor%5D&cauthor=true&cauthor_uid=20212336) | India | 20212336 | 95 | Retrospective |
| 29 | 2000-2006 | 2010 | Verma S | India | 20061765 | 159 | Retrospective |
| 30 | 2004-2006 | 2010 | Mengo DM |  | 20601792 | 100 | Retrospective |
| 31 | 2004 | 2009 | [Muyembe-Tamfum JJ](https://www.ncbi.nlm.nih.gov/pubmed/?term=Muyembe-Tamfum%20JJ%5BAuthor%5D&cauthor=true&cauthor_uid=19174300) | DRC | 19174300 | 11 | Retrospective |
| 32 | 2008 | 2009 | Kumar Y | India | 19762961 | 50 | Retrospective |
| 33 |  | 2009 | Yanagi D | Indonesia | 19631095 | 17 | Retrospective |
| 34 | 1999-2006 | 2009 | Lynch MF | Travel Associated | 19706859 | 2016 | Retrospective |
| 35 | 2005 | 2008 | [Mirza SH](https://www.ncbi.nlm.nih.gov/pubmed/?term=Mirza%20SH%5BAuthor%5D&cauthor=true&cauthor_uid=18452661) | Pakistan | 18452661 | 32 | Retrospective |
| 36 | 2008 | 2008 | [Prajapati B](https://www.ncbi.nlm.nih.gov/pubmed/?term=Prajapati%20B%5BAuthor%5D&cauthor=true&cauthor_uid=19558061) | Nepal | 19558061 | 195 | Retrospective |
| 37 | 2007 | 2008 | Al-Sanouri TM | Jordan | 19741292 | 48 | Retrospective |
| 38 | 1992 | 2007 | [Rodrigues C](https://www.ncbi.nlm.nih.gov/pubmed/?term=Rodrigues%20C%5BAuthor%5D&cauthor=true&cauthor_uid=1307533) | India | 1307533 | 74 | Retrospective |
| 39 | 2004 | 2007 | [Joshi S](https://www.ncbi.nlm.nih.gov/pubmed/?term=Joshi%20S%5BAuthor%5D&cauthor=true&cauthor_uid=16950486) | India | 16950486 | 25 | Retrospective |
| 40 | 2007 | 2007 | [Parry CM](https://www.ncbi.nlm.nih.gov/pubmed/?term=Parry%20CM%5BAuthor%5D&cauthor=true&cauthor_uid=17145784) | Vietnam | 17145784 | 187 | RCT |
| 41 | 2003 | 2007 | [Bhatta DR](https://www.ncbi.nlm.nih.gov/pubmed/?term=Bhatta%20DR%5BAuthor%5D&cauthor=true&cauthor_uid=17576218) | Nepal | 17576218 | 16 | Retrospective |
| 42 | 2004-2005 | 2007 | Akinyemi KO | Nigeria | 18330069 | 89 | Prospective |
| 43 | 2005 | 2007 | [Tamang MD](https://www.ncbi.nlm.nih.gov/pubmed/?term=Tamang%20MD%5BAuthor%5D&cauthor=true&cauthor_uid=17629465) | Nepal | 17629465 | 93 | Retrospective |
| 44 | 2002 | 2007 | Capoor MR | India | 17873998 | 178 | Retrospective |
| 45 | 2003 | 2007 | [Banerjee A](https://www.ncbi.nlm.nih.gov/pubmed/?term=Banerjee%20A%5BAuthor%5D&cauthor=true&cauthor_uid=27408039) | India | 27408039 | 60 | Retrospective |
| 46 |  | 2007 | Khanal B | Nepal | 17615907 | 132 | Retrospective |
| 47 | 1993-2005 | 2007 | Chau TT | Vietnam, Indonesia, Laos,  Pakistan,  Nepal,  China,  India,  Bangladesh | 17908946 | 1774 | Retrospective |
| 48 | 1997-2004 | 2007 | Akinyemi KO | Nigeria | 18087113 | 274 | Retrospective |
| 49 | 2004-2005 | 2006 | [Manchanda V](https://www.ncbi.nlm.nih.gov/pubmed/?term=Manchanda%20V%5BAuthor%5D&cauthor=true&cauthor_uid=16687859) | India | 16687859 | 56 | Retrospective |
| 50 | 2006 | 2006 | [Ray P](https://www.ncbi.nlm.nih.gov/pubmed/?term=Ray%20P%5BAuthor%5D&cauthor=true&cauthor_uid=16926465) | India | 16926465 | 70 | Cross-Sectional |
| 51 | 1999-2004 | 2006 | Mohanty S | India | 16476168 | 629 | Retrospective |
| 52 | 1989-2002 | 2006 | Rahman | Bangladesh | 16490150 | 3927 | Retrospective |
| 53 | 2001-2004 | 2006 | Lakshmi V | India | 16505555 | 60 | Retrospective |
| 54 | 2000-2001 | 2005 | [Brooks WA](https://www.ncbi.nlm.nih.gov/pubmed/?term=Brooks%20WA%5BAuthor%5D&cauthor=true&cauthor_uid=15752457) | Bangladesh | 15752457 | 49 | Prospective |
| 55 | 2003-2004 | 2005 | [Dutta S](https://www.ncbi.nlm.nih.gov/pubmed/?term=Dutta%20S%5BAuthor%5D&cauthor=true&cauthor_uid=15793167) | India | 15793167 | 379 | Retrospective |
| 56 | 2004 | 2005 | [Senthilkumar B](https://www.ncbi.nlm.nih.gov/pubmed/?term=Senthilkumar%20B%5BAuthor%5D&cauthor=true&cauthor_uid=15928436) | India | 15928436 | 6 | Retrospective |
| 57 | 2002 | 2004 | [Madhulika U](https://www.ncbi.nlm.nih.gov/pubmed/?term=Madhulika%20U%5BAuthor%5D&cauthor=true&cauthor_uid=15347861) | India | 15347861 | 157 | Cross Sectional |
| 58 | 2002 | 2004 | [Mamun KZ](https://www.ncbi.nlm.nih.gov/pubmed/?term=Mamun%20KZ%5BAuthor%5D&cauthor=true&cauthor_uid=16240978) | Bangladesh | 16240978 | 30 | Retrospective |
| 59 | 1997-2001 | 2002 | Gautam V | India | 12585971 | 436 | Retrospective |
| 60 | 2001-2003 | 2005 | Kadhiravan T | India | 15904505 | 50 | Retrospective |
| 61 | 1977 | 1977 | [Butler T](https://www.ncbi.nlm.nih.gov/pubmed/?term=Butler%20T%5BAuthor%5D&cauthor=true&cauthor_uid=324398) | Vietnam | 324398 | 87 | Retrospective |
| 62 | 1973 | 1973 | [Lawrence RM](https://www.ncbi.nlm.nih.gov/pubmed/?term=Lawrence%20RM%5BAuthor%5D&cauthor=true&cauthor_uid=4572522) | Travel Associated | 4572522 | 1 | Case Report |
| 63 | 1973 | 1973 | [Overturf G](https://www.ncbi.nlm.nih.gov/pubmed/?term=Overturf%20G%5BAuthor%5D&cauthor=true&cauthor_uid=4763412) | Travel Associated | 4763412 | 28 | Retrospective |
