# Supplementary material for: A systematic review of antimicrobial resistance in Salmonella enterica serovar Typhi, the etiological agent of typhoid
Source: PLoS Negl Trop Dis. 2018 Oct 11;12(10):e0006779. doi: 10.1371/journal.pntd.0006779 (PMC6198998; doi:10.1371/journal.pntd.0006779)
Supplement: S2 Table — (DOCX) [file pntd.0006779.s002.docx]

| **Supplementary Table 2: Characteristics included publications included in the molecular analysis of AMR** | | | | | | |
| --- | --- | --- | --- | --- | --- | --- |
| No. | Year of Publication | Author | Country/Region of study | PMID | Number of isolates | Genotyping method |
| 1 | 2018 | Klemm E | Pakistan | 29463654 | 80 | WGS |
| 2 | 2017 | [Ramachandran A](https://www.ncbi.nlm.nih.gov/pubmed/?term=Ramachandran%20A%5BAuthor%5D&cauthor=true&cauthor_uid=29207706) | India | 29207706 | 2 | PCR |
| 3 | 2017 | [Das S](https://www.ncbi.nlm.nih.gov/pubmed/?term=Das%20S%5BAuthor%5D&cauthor=true&cauthor_uid=27916384) | India | 27916384 | 165 | PGFE |
| 4 | 2016 | [Gopal M](https://www.ncbi.nlm.nih.gov/pubmed/?term=Gopal%20M%5BAuthor%5D&cauthor=true&cauthor_uid=27630841) | India | 27630841 | 131 | PCR |
| 5 | 2016 | Misra | India | 27618918 | 100 | PCR |
| 6 | 2016 | Ragupathi D | India | 27530999 | 1 | WGS |
| 7 | 2016 | [Elumalai S](https://www.ncbi.nlm.nih.gov/pubmed/?term=Elumalai%20S%5BAuthor%5D&cauthor=true&cauthor_uid=27166067) | India | 27166067 | 1 | PCR |
| 8 | 2016 | [Al-Emran HM](https://www.ncbi.nlm.nih.gov/pubmed/?term=Al-Emran%20HM%5BAuthor%5D&cauthor=true&cauthor_uid=26933020) | Africa (TSAP) | 26933020 | 11 | WGS |
| 9 | 2016 | Thanh D | Nepal | 26974227 | 78 | WGS |
| 10 | 2016 | Wong V | Nigeria | 27657909 | 128 | WGS |
| 11 | 2015 | [García-Fernández A](https://www.ncbi.nlm.nih.gov/pubmed/?term=Garc%C3%ADa-Fern%C3%A1ndez%20A%5BAuthor%5D&cauthor=true&cauthor_uid=26121266) | Italy | 26121266 | 17 | PCR |
| 12 | 2015 | [Nüesch-Inderbinen M](https://www.ncbi.nlm.nih.gov/pubmed/?term=N%C3%BCesch-Inderbinen%20M%5BAuthor%5D&cauthor=true&cauthor_uid=25963025) | Switzerland | 25963025 | 83 | PCR |
| 13 | 2015 | [Akinyemi KO](https://www.ncbi.nlm.nih.gov/pubmed/?term=Akinyemi%20KO%5BAuthor%5D&cauthor=true&cauthor_uid=25999745) | Nigeria | 25999745 | 11 | PCR |
| 14 | 2015 | [Ceyssens PJ](https://www.ncbi.nlm.nih.gov/pubmed/?term=Ceyssens%20PJ%5BAuthor%5D&cauthor=true&cauthor_uid=25385108) | Belgium | 25385108 | 62 | PCR |
| 15 | 2015 | Wong V | Global | 25961941 | 1832 | WGS |
| 16 | 2014 | [Chiou CS](https://www.ncbi.nlm.nih.gov/pubmed/?term=Chiou%20CS%5BAuthor%5D&cauthor=true&cauthor_uid=25136011) | Bangladesh, Indonesia, Taiwan, Vietnam | 25136011 | 38 | PCR |
| 17 | 2014 | Dutta S | India | 25098613 | 18 | PCR |
| 18 | 2014 | [González-López JJ](https://www.ncbi.nlm.nih.gov/pubmed/?term=Gonz%C3%A1lez-L%C3%B3pez%20JJ%5BAuthor%5D&cauthor=true&cauthor_uid=25340972) | Guatemala | 25340972 | 1 | PCR |
| 19 | 2014 | [Dahiya S](https://www.ncbi.nlm.nih.gov/pubmed/?term=Dahiya%20S%5BAuthor%5D&cauthor=true&cauthor_uid=25027085) | India | 25027085 | 18 | PCR |
| 20 | 2014 | Saleh FO | Egypt | 24820472 | 4 | PCR |
| 21 | 2014 | [Geetha VK](https://www.ncbi.nlm.nih.gov/pubmed/?term=Geetha%20VK%5BAuthor%5D&cauthor=true&cauthor_uid=24399384) | India | 24399384 | 36 | PCR |
| 22 | 2013 | Lee CJ | Taiwan | 23465712 | 5 | PFGE |
| 23 | 2013 | [Jain S](https://www.ncbi.nlm.nih.gov/pubmed/?term=Jain%20S%5BAuthor%5D&cauthor=true&cauthor_uid=24240035) | India | 24240035 | 266 | PCR |
| 24 | 2012 | [Lunguya O](https://www.ncbi.nlm.nih.gov/pubmed/?term=Lunguya%20O%5BAuthor%5D&cauthor=true&cauthor_uid=23166855) | Congo | 23166855 | 31 | PFGE |
| 25 | 2012 | [Vlieghe ER](https://www.ncbi.nlm.nih.gov/pubmed/?term=Vlieghe%20ER%5BAuthor%5D&cauthor=true&cauthor_uid=23272255) | Cambodia | 23272255 | 59 | PFGE |
| 26 | 2012 | Emary K | Cambodia | 23122884 | 102 | PCR |
| 27 | 2012 | [Thamizhmani R](https://www.ncbi.nlm.nih.gov/pubmed/?term=Thamizhmani%20R%5BAuthor%5D&cauthor=true&cauthor_uid=22885270) | India | 22885270 | 6 | PCR |
| 28 | 2012 | [Tatavarthy A](https://www.ncbi.nlm.nih.gov/pubmed/?term=Tatavarthy%20A%5BAuthor%5D&cauthor=true&cauthor_uid=22649021) | USA | 22649021 | 16 | PCR |
| 29 | 2012 | [Acharya D](https://www.ncbi.nlm.nih.gov/pubmed/?term=Acharya%20D%5BAuthor%5D&cauthor=true&cauthor_uid=22627312) | Nepal | 22627312 | 11 | PCR |
| 30 | 2012 | Ahmed D | Bangladesh | 22442289 | 1 | PCR |
| 31 | 2012 | [Koirala KD](https://www.ncbi.nlm.nih.gov/pubmed/?term=Koirala%20KD%5BAuthor%5D&cauthor=true&cauthor_uid=22371897) | Nepal | 22371897 | 1 | PCR |
| 32 | 2012 | [Kumarasamy K](https://www.ncbi.nlm.nih.gov/pubmed/?term=Kumarasamy%20K%5BAuthor%5D&cauthor=true&cauthor_uid=22146877) | India | 22146877 | 1 | PCR |
| 33 | 2011 | [Accou-Demartin M](https://www.ncbi.nlm.nih.gov/pubmed/?term=Chau%20TT%5BAuthor%5D&cauthor=true&cauthor_uid=17908946) | France | 21749778 | 11 | PCR |
| 34 | 2011 | [Hassing RJ](https://www.ncbi.nlm.nih.gov/pubmed/?term=Chau%20TT%5BAuthor%5D&cauthor=true&cauthor_uid=17908946) | Netherlands | 21227657 | 11 | PCR |
| 35 | 2010 | [Mohanty S](https://www.ncbi.nlm.nih.gov/pubmed/?term=Mohanty%20S%5BAuthor%5D&cauthor=true&cauthor_uid=20828458) | India | 20828458 | 1 | PCR |
| 36 | 2010 | [Gaborieau V](https://www.ncbi.nlm.nih.gov/pubmed/?term=Gaborieau%20V%5BAuthor%5D&cauthor=true&cauthor_uid=20724089) | France | 20724089 | 1 | PCR |
| 37 | 2010 | [Nath G](https://www.ncbi.nlm.nih.gov/pubmed/?term=Nath%20G%5BAuthor%5D&cauthor=true&cauthor_uid=20188522) | India | 20188522 | 90 | PCR |
| 38 | 2010 | [Morita M](https://www.ncbi.nlm.nih.gov/pubmed/?term=Morita%20M%5BAuthor%5D&cauthor=true&cauthor_uid=20585124) | Japan | 20585124 | 1 | PCR |
| 39 | 2010 | Wu W | China | 20113512 | 25 | PCR |
| 40 | 2010 | [Dimitrov T](https://www.ncbi.nlm.nih.gov/pubmed/?term=Dimitrov%20T%5BAuthor%5D&cauthor=true&cauthor_uid=19889623) | Kuwait | 19889623 | 26 | CT |
| 41 | 2009 | [Pfeifer Y](https://www.ncbi.nlm.nih.gov/pubmed/?term=Pfeifer%20Y%5BAuthor%5D&cauthor=true&cauthor_uid=19788837) | Germany | 19788837 | 1 | PCR |
| 42 | 2009 | [Yanagi D](https://www.ncbi.nlm.nih.gov/pubmed/?term=Yanagi%20D%5BAuthor%5D&cauthor=true&cauthor_uid=19631095) | Indonesia | 19631095 | 17 | PCR |
| 43 | 2009 | [Yoon HJ](https://www.ncbi.nlm.nih.gov/pubmed/?term=Shanahan%20PM%5BAuthor%5D&cauthor=true&cauthor_uid=10722124) | Korea | 19259362 | 1 | PCR |
| 44 | 2009 | [Dimitrov T](https://www.ncbi.nlm.nih.gov/pubmed/?term=Shanahan%20PM%5BAuthor%5D&cauthor=true&cauthor_uid=10722124) | Kuwait | 18971360 | 2 | PCR |
| 45 | 2009 | [Capoor MR](https://www.ncbi.nlm.nih.gov/pubmed/?term=Shanahan%20PM%5BAuthor%5D&cauthor=true&cauthor_uid=10722124) | India | 18687156 | 14 | PCR |
| 46 | 2008 | [Dashti AA](https://www.ncbi.nlm.nih.gov/pubmed/?term=Shanahan%20PM%5BAuthor%5D&cauthor=true&cauthor_uid=10722124) | Kuwait | 18606582 | 25 | PCR |
| 47 | 2008 | [Rotimi VO](https://www.ncbi.nlm.nih.gov/pubmed/?term=Rotimi%20VO%5BAuthor%5D&cauthor=true&cauthor_uid=18566147) | Kuwait | 18566147 | 2 | PCR |
| 48 | 2008 | [Dutta S](https://www.ncbi.nlm.nih.gov/pubmed/?term=Dutta%20S%5BAuthor%5D&cauthor=true&cauthor_uid=18280709) | India | 18280709 | 2 | PCR |
| 49 | 2008 | Al-Sanouri | Jordan | 19741292 | 45 | PCR |
| 50 | 2007 | [Chau TT](https://www.ncbi.nlm.nih.gov/pubmed/?term=Chau%20TT%5BAuthor%5D&cauthor=true&cauthor_uid=17908946) | India | 17908946 | 23 | PCR |
|  | 2007 | Chau TT | Pakistan | 17908946 | 34 | PCR |
|  | 2007 | Chau TT | Vietnam | 17908946 | 118 | PCR |
| 51 | 2007 | [Capoor MR](https://www.ncbi.nlm.nih.gov/pubmed/?term=Capoor%20MR%5BAuthor%5D&cauthor=true&cauthor_uid=17873998) | India | 17873998 | 12 | PCR |
| 52 | 2007 | [Tamang MD](https://www.ncbi.nlm.nih.gov/pubmed/?term=Tamang%20MD%5BAuthor%5D&cauthor=true&cauthor_uid=17629465) | Nepal | 17629465 | 93 | PCR |
| 53 | 2006 | [Gaind R](https://www.ncbi.nlm.nih.gov/pubmed/?term=Gaind%20R%5BAuthor%5D&cauthor=true&cauthor_uid=17071955) | India | 17071955 | 8 | PCR |
| 54 | 2006 | [Shirakawa T](https://www.ncbi.nlm.nih.gov/pubmed/?term=Shirakawa%20T%5BAuthor%5D&cauthor=true&cauthor_uid=16466897) | Nepal | 16466897 | 30 | PCR |
| 55 | 2004 | [Lee K](https://www.ncbi.nlm.nih.gov/pubmed/?term=Lee%20K%5BAuthor%5D&cauthor=true&cauthor_uid=15504831) | South Korea | 15504831 | 11 | PCR |
| 57 | 2004 | [Renuka K](https://www.ncbi.nlm.nih.gov/pubmed/?term=Renuka%20K%5BAuthor%5D&cauthor=true&cauthor_uid=15256030) | India | 15256030 | 52 | PCR |
| 58 | 2002 | [Mills-Robertson F](https://www.ncbi.nlm.nih.gov/pubmed/?term=Mills-Robertson%20F%5BAuthor%5D&cauthor=true&cauthor_uid=12399042) | Ghana | 12399042 | 21 | PCR |
| 59 | 2000 | [Shanahan PM](https://www.ncbi.nlm.nih.gov/pubmed/?term=Shanahan%20PM%5BAuthor%5D&cauthor=true&cauthor_uid=10722124) | Pakistan | 10722124 | 147 | CT |
|  | 2000 | Shanahan PM | Bangladesh | 10722124 | 30 | CT |
|  | 2000 | Shanahan PM | Kuwait | 10722124 | 8 | CT |
|  | 2000 | Shanahan PM | Malaysia | 10722124 | 6 | CT |
|  | 2000 | Shanahan PM | India | 10722124 | 2 | CT |
|  | 1998 | Shanahan PM | India | 9620383 | 20 | CT |
| 60 | 1997 | [Wain J](https://www.ncbi.nlm.nih.gov/pubmed/?term=Wain%20J%5BAuthor%5D&cauthor=true&cauthor_uid=9431387) | Vietnam | 9431387 | 20 | CT |
| 61 | 1996 | [Panigrahi D](https://www.ncbi.nlm.nih.gov/pubmed/?term=Panigrahi%20D%5BAuthor%5D&cauthor=true&cauthor_uid=8765450) | Kuwait | 8765450 | 91 | CT |

PMID – PunMed Identifier

WGS – Whole genome sequencing

PCR – Polymerase chain reaction

PFGE – Pulse field gel electrophoresis

CT – Conjugational transfer using *E. Coli*.
