# Supplementary material for: A systematic review of antimicrobial resistance in Salmonella enterica serovar Typhi, the etiological agent of typhoid
Source: PLoS Negl Trop Dis. 2018 Oct 11;12(10):e0006779. doi: 10.1371/journal.pntd.0006779 (PMC6198998; doi:10.1371/journal.pntd.0006779)
Supplement: S3 Table — (DOCX) [file pntd.0006779.s003.docx]

| **Supplementary Table 3: Risk of bias assessment** | | | | | | | | | | | | | |
| --- | --- | --- | --- | --- | --- | --- | --- | --- | --- | --- | --- | --- | --- |
| **Objective 1: Phenotypic trends of AMR in typhoid fever** | | | | | | | | | | | | | |
| **Included Studies** | | | Risk of bias domains (QUIPS) | | | | | Risk of bias domains (JBI) | | | | | |
|  |  |  | **Study population** | **Measurement of outcome(s)** | **Study Attrition** | **Statistical analysis and reporting** | **Risk of Bias** | **Population description** | **Prospective sampling** | **Microbiologic**  **methods** | **Sample size**  **>200** | **Performance standards used** | **Risk of Bias** |
| First author | Publication year | PubMed IDentifier | Domain 1 | Domain 2 | Domain 3 | Domain 4 |  | Q1 | Q2 | Q3 | Q4 | Q5 |  |
| Ali A | 2017 | 28303985 | Low | Low | Low | Low | **Low** | Yes | No | Yes | Yes | Yes | **No** |
| Harichandran D | 2017 | 28352198 | Low | Low | Low | Moderate | **Low** | Yes | No | Yes | Yes | Yes | **No** |
| Osbourne LG | 2016 | 26243802 | Low | Low | NA | Low | **Low** | Yes | NA | Yes | Yes | Yes | **Yes** |
| [Sharvani R](https://www.ncbi.nlm.nih.gov/pubmed/?term=Sharvani%20R%5BAuthor%5D&cauthor=true&cauthor_uid=27437211) | 2013 | 27437211 | Low | Low | Low | Low | **Low** | Yes | No | Yes | Yes | Yes | **No** |
| Misra R | 2016 | 25979527 | Low | Low | Low | Low | **Low** | Yes | No | Yes | Yes | Yes | **No** |
| [Khanam F](https://www.ncbi.nlm.nih.gov/pubmed/?term=Khanam%20F%5BAuthor%5D&cauthor=true&cauthor_uid=25849611) | 2015 | 25849611 | Low | Low | Low | Low | **Low** | Yes | No | Yes | Yes | Yes | **No** |
| [Mahende C](https://www.ncbi.nlm.nih.gov/pubmed/?term=Mahende%20C%5BAuthor%5D&cauthor=true&cauthor_uid=26138060) | 2015 | 26138060 | Low | Low | Low | Low | **Low** | Yes | Yes | Yes | Yes | Yes | **No** |
| [Narain U](https://www.ncbi.nlm.nih.gov/pubmed/?term=Narain%20U%5BAuthor%5D&cauthor=true&cauthor_uid=26388636) | 2015 | 26388636 | Low | Low | Low | Low | **Low** | Yes | No | Yes | Yes | Yes | **No** |
| Nüesch-Inderbinen M | 2015 | 25963025 | Moderate | Low | Low | Low | **Low** | No | No | Yes | Yes | Yes | **No** |
| [Jessica Maltha](https://www.ncbi.nlm.nih.gov/pubmed/?term=Maltha%20J%5BAuthor%5D&cauthor=true&cauthor_uid=24551225) | 2014 | 24551225 | Low | Low | Low | Low | **Low** | Yes | Yes | Yes | Yes | Yes | **No** |
| [Srirangaraj S](https://www.ncbi.nlm.nih.gov/pubmed/?term=Srirangaraj%20S%5BAuthor%5D&cauthor=true&cauthor_uid=24817913) | 2014 | 24817913 | Low | Low | Low | Low | **Low** | Yes | No | Yes | Yes | Yes | **No** |
| [Chiou CS](https://www.ncbi.nlm.nih.gov/pubmed/?term=Chiou%20CS%5BAuthor%5D&cauthor=true&cauthor_uid=25136011) | 2014 | 25136011 | Low | Low | Low | Low | **Low** | Yes | No | Yes | Yes | Yes | **No** |
| [Chand HJ](https://www.ncbi.nlm.nih.gov/pubmed/?term=Chand%20HJ%5BAuthor%5D&cauthor=true&cauthor_uid=25390062) | 2014 | 25390062 | Low | Low | Low | Low | **Low** | Yes | Yes | Yes | Yes | Yes | **No** |
| [Isendahl J](https://www.ncbi.nlm.nih.gov/pubmed/?term=Isendahl%20J%5BAuthor%5D&cauthor=true&cauthor_uid=25526763) | 2014 | 25526763 | Low | Low | Low | Low | **Low** | Yes | Yes | Yes | Yes | Yes | **Yes** |
| [Dahiya S](https://www.ncbi.nlm.nih.gov/pubmed/?term=Dahiya%20S%5BAuthor%5D&cauthor=true&cauthor_uid=28303820) | 2014 | 28303820 | Low | Low | Low | Low | **Low** | Yes | No | Yes | Yes | Yes | **No** |
| [Choudhary](https://www.ncbi.nlm.nih.gov/pubmed/?term=Choudhary%20A%5BAuthor%5D&cauthor=true&cauthor_uid=23703350) A | 2013 | 23703350 | Low | Low | Low | Low | **Low** | Yes | No | Yes | Yes | Yes | **No** |
| Sultan BA | 2013 | 23905456 | Low | Low | NA | Low | **Low** | No | NA | Yes | No | Yes | **Yes** |
| [Vlieghe E](https://www.ncbi.nlm.nih.gov/pubmed/?term=Vlieghe%20E%5BAuthor%5D&cauthor=true&cauthor_uid=24094060) | 2013 | 24094060 | Low | Low | Low | Low | **Low** | Yes | No | Yes | No | Yes | **No** |
| [Venkatesh BM](https://www.ncbi.nlm.nih.gov/pubmed/?term=Venkatesh%20BM%5BAuthor%5D&cauthor=true&cauthor_uid=24441263) | 2013 | 24441263 | Low | Low | Low | Low | **Low** | Yes | No | Yes | Yes | Yes | **No** |
| Gupta V | 2013 | 24043999 | Low | Low | Low | Low | **Low** | Yes | No | Yes | Yes | Yes | **No** |
| Jain S | 2013 | 24240035 | Low | Low | Low | Low | **Low** | Yes | No | Yes | Yes | Yes | **No** |
| [Lutterloh E](https://www.ncbi.nlm.nih.gov/pubmed/?term=Lutterloh%20E%5BAuthor%5D&cauthor=true&cauthor_uid=22357702) | 2012 | 22357702 | Moderate | Low | Low | Low | **Low** | Yes | No | Yes | No | Yes | **No** |
| [Olut AI](https://www.ncbi.nlm.nih.gov/pubmed/?term=Olut%20AI%5BAuthor%5D&cauthor=true&cauthor_uid=22399179) | 2012 | 22399179 | Low | Low | NA | Low | **Low** | Yes | NA | Yes | No | Yes | **Yes** |
| [Acharya D](https://www.ncbi.nlm.nih.gov/pubmed/?term=Acharya%20D%5BAuthor%5D&cauthor=true&cauthor_uid=22627312) | 2012 | 22627312 | Low | Low | Low | Low | **Low** | Yes | No | Yes | No | Yes | **No** |
| [Kumar Y](https://www.ncbi.nlm.nih.gov/pubmed/?term=Kumar%20Y%5BAuthor%5D&cauthor=true&cauthor_uid=21444993) | 2011 | 21444993 | Low | Low | Low | Low | **Low** | Yes | No | Yes | No | Yes | **No** |
| Adhikary | 2011 | 22234135 | Low | Low | NA | Low | Low | Yes | NA | Yes | No | Yes | **Yes** |
| Gross U | 2011 | 22000360 | Low | Low | Low | Low | **Low** | Yes | No | Yes | No | Yes | **No** |
| [Nagshetty K](https://www.ncbi.nlm.nih.gov/pubmed/?term=Nagshetty%20K%5BAuthor%5D&cauthor=true&cauthor_uid=20212336) | 2010 | 20212336 | Low | Low | Low | Low | **Low** | Yes | No | Yes | No | Yes | **No** |
| Verma S | 2010 | 20061765 | Low | Low | Low | Low | **Low** | Yes | No | Yes | No | Yes | **No** |
| Mengo DM | 2010 | 20601792 | Low | Low | Low | Low | **Low** | Yes | No | Yes | No | Yes | **No** |
| [Muyembe-Tamfum JJ](https://www.ncbi.nlm.nih.gov/pubmed/?term=Muyembe-Tamfum%20JJ%5BAuthor%5D&cauthor=true&cauthor_uid=19174300) | 2009 | 19174300 | Low | Low | Low | Low | **Low** | Yes | No | Yes | No | Yes | **No** |
| Kumar Y | 2009 | 19762961 | Low | Low | Low | Low | **Low** | Yes | No | Yes | No | Yes | **No** |
| Yanagi D | 2009 | 19631095 | Low | Low | Low | Low | **Low** | Yes | No | Yes | No | Yes | **No** |
| Lynch MF | 2009 | 19706859 | Moderate | Low | Low | Low | **Low** | Yes | No | Yes | Yes | Yes | **No** |
| [Mirza SH](https://www.ncbi.nlm.nih.gov/pubmed/?term=Mirza%20SH%5BAuthor%5D&cauthor=true&cauthor_uid=18452661) | 2008 | 18452661 | Low | Low | Low | Low | **Low** | Yes | No | Yes | No | Yes | **No** |
| [Prajapati B](https://www.ncbi.nlm.nih.gov/pubmed/?term=Prajapati%20B%5BAuthor%5D&cauthor=true&cauthor_uid=19558061) | 2008 | 19558061 | Low | Low | Low | Low | **Low** | Yes | No | Yes | No | Yes | **No** |
| Al-Sanouri TM | 2008 | 19741292 | Low | Low | Low | Low | **Low** | Yes | No | Yes | No | Yes | **No** |
| [Rodrigues C](https://www.ncbi.nlm.nih.gov/pubmed/?term=Rodrigues%20C%5BAuthor%5D&cauthor=true&cauthor_uid=1307533) | 2007 | 1307533 | Low | Low | Low | Low | **Low** | Yes | No | Yes | No | Yes | **No** |
| [Joshi S](https://www.ncbi.nlm.nih.gov/pubmed/?term=Joshi%20S%5BAuthor%5D&cauthor=true&cauthor_uid=16950486) | 2007 | 16950486 | Low | Low | Low | Low | **Low** | Yes | No | Yes | No | Yes | **No** |
| [Parry CM](https://www.ncbi.nlm.nih.gov/pubmed/?term=Parry%20CM%5BAuthor%5D&cauthor=true&cauthor_uid=17145784) | 2007 | 17145784 | Low | Low | Low | Low | **Low** | Yes | Yes | Yes | No | Yes | **No** |
| [Bhatta DR](https://www.ncbi.nlm.nih.gov/pubmed/?term=Bhatta%20DR%5BAuthor%5D&cauthor=true&cauthor_uid=17576218) | 2007 | 17576218 | Low | Low | Low | Low | **Low** | Yes | No | Yes | No | Yes | **No** |
| Akinyemi KO | 2007 | 18330069 | Low | Low | Low | Low | **Low** | Yes | Yes | Yes | No | Yes | **No** |
| [Tamang MD](https://www.ncbi.nlm.nih.gov/pubmed/?term=Tamang%20MD%5BAuthor%5D&cauthor=true&cauthor_uid=17629465) | 2007 | 17629465 | Low | Low | Low | Low | **Low** | Yes | No | Yes | No | Yes | **No** |
| Capoor MR | 2007 | 17873998 | Low | Low | Low | Low | **Low** | Yes | No | Yes | No | Yes | **No** |
| [Banerjee A](https://www.ncbi.nlm.nih.gov/pubmed/?term=Banerjee%20A%5BAuthor%5D&cauthor=true&cauthor_uid=27408039) | 2007 | 27408039 | Low | Low | Low | Low | **Low** | Yes | No | Yes | No | Yes | **No** |
| Khanal B | 2007 | 17615907 | Low | Low | Low | Low | **Low** | Yes | No | Yes | No | Yes | **No** |
| Chau TT | 2007 | 17908946 | Low | Low | Low | Low | **Low** | Yes | No | Yes | Low | Yes | **No** |
| Akinyemi KO | 2007 | 18087113 | Low | Low | Low | Low | **Low** | Yes | No | Yes | No | Yes | **No** |
| [Manchanda V](https://www.ncbi.nlm.nih.gov/pubmed/?term=Manchanda%20V%5BAuthor%5D&cauthor=true&cauthor_uid=16687859) | 2006 | 16687859 | Low | Low | Low | Low | **Low** | Yes | No | Yes | No | Yes | **No** |
| [Ray P](https://www.ncbi.nlm.nih.gov/pubmed/?term=Ray%20P%5BAuthor%5D&cauthor=true&cauthor_uid=16926465) | 2006 | 16926465 | Low | Low | Low | Low | **Low** | Yes | No | Yes | No | Yes | **No** |
| Mohanty S | 2006 | 16476168 | Low | Low | Low | Low | **Low** | Yes | No | Yes | Low | Yes | **No** |
| Rahman | 2006 | 16490150 | Low | Low | Low | Low | **Low** | Yes | No | Yes | Low | Yes | **No** |
| Lakshmi V | 2006 | 16505555 | Low | Low | Low | Low | **Low** | Yes | No | Yes | No | Yes | **No** |
| [Brooks WA](https://www.ncbi.nlm.nih.gov/pubmed/?term=Brooks%20WA%5BAuthor%5D&cauthor=true&cauthor_uid=15752457) | 2005 | 15752457 | Low | Low | Low | Low | **Low** | Yes | No | Yes | No | Yes | **No** |
| [Dutta S](https://www.ncbi.nlm.nih.gov/pubmed/?term=Dutta%20S%5BAuthor%5D&cauthor=true&cauthor_uid=15793167) | 2005 | 15793167 | Low | Low | Low | Low | **Low** | Yes | No | Yes | Low | Yes | **No** |
| [Senthilkumar B](https://www.ncbi.nlm.nih.gov/pubmed/?term=Senthilkumar%20B%5BAuthor%5D&cauthor=true&cauthor_uid=15928436) | 2005 | 15928436 | Low | Low | Low | Low | **Low** | Yes | No | Yes | No | Yes | **No** |
| [Madhulika U](https://www.ncbi.nlm.nih.gov/pubmed/?term=Madhulika%20U%5BAuthor%5D&cauthor=true&cauthor_uid=15347861) | 2004 | 15347861 | Low | Low | Low | Low | **Low** | Yes | No | Yes | No | Yes | **No** |
| [Mamun KZ](https://www.ncbi.nlm.nih.gov/pubmed/?term=Mamun%20KZ%5BAuthor%5D&cauthor=true&cauthor_uid=16240978) | 2004 | 16240978 | Low | Low | Low | Low | **Low** | Yes | No | Yes | No | Yes | **No** |
| Gautam V | 2002 | 12585971 | Low | Low | Low | Low | **Low** | Yes | No | Yes | Low | Yes | **No** |
| Kadhiravan T | 2005 | 15904505 | Low | Low | Low | Low | **Low** | Yes | No | Yes | No | Yes | **No** |
| [Butler T](https://www.ncbi.nlm.nih.gov/pubmed/?term=Butler%20T%5BAuthor%5D&cauthor=true&cauthor_uid=324398) | 1977 | 324398 | Low | Low | Low | Low | **Low** | Yes | No | Yes | No | Yes | **No** |
| [Lawrence RM](https://www.ncbi.nlm.nih.gov/pubmed/?term=Lawrence%20RM%5BAuthor%5D&cauthor=true&cauthor_uid=4572522) | 1973 | 4572522 | Low | Low | NA | Low | **Low** | No | NA | Yes | No | Yes | **Yes** |
| [Overturf G](https://www.ncbi.nlm.nih.gov/pubmed/?term=Overturf%20G%5BAuthor%5D&cauthor=true&cauthor_uid=4763412) | 1973 | 4763412 | Low | Low | Low | Low | **Low** | Yes | No | Yes | No | Yes | **No** |

| **Objective 2: Molecular determinants of AMR in typhoid fever** | | | | | | | | | | | | | |
| --- | --- | --- | --- | --- | --- | --- | --- | --- | --- | --- | --- | --- | --- |
| **Included Studies** | | | Risk of bias domains (QUIPS)  (Hayden et al. 2013) | | | | | Risk of bias domains (JBI) | | | | | |
|  |  |  | **Study population** | **Measurement of outcome(s)** | **Study Attrition** | **Statistical analysis and reporting** | **Risk of Bias** | **Population description** | **Prospective sampling** | **Bacteriologic culture**  **Methods** | **Sample size over 100** | **Genotyping method** | **Risk of Bias** |
| First author | Publication year | PubMed IDentifier | Domain 1 | Domain 2 | Domain 3 | Domain 4 |  | Q1 | Q2 | Q3 | Q4 | Q5 |  |
| Klemm E | 2018 | 29463654 | Low | Low | Low | Low | **Low** | Yes | No | Yes | No | Yes | **No** |
| [Ramachandran A](https://www.ncbi.nlm.nih.gov/pubmed/?term=Ramachandran%20A%5BAuthor%5D&cauthor=true&cauthor_uid=29207706) | 2017 | 29207706 | Low | Low | Moderate | Low | **Low** | Yes | No | Yes | No | No | **No** |
| [Das S](https://www.ncbi.nlm.nih.gov/pubmed/?term=Das%20S%5BAuthor%5D&cauthor=true&cauthor_uid=27916384) | 2017 | 27916384 | Low | Low | Low | Low | **Low** | Yes | No | Yes | Yes | No | **No** |
| [Gopal M](https://www.ncbi.nlm.nih.gov/pubmed/?term=Gopal%20M%5BAuthor%5D&cauthor=true&cauthor_uid=27630841) | 2016 | 27630841 | Low | Low | Low | Low | **Low** | Yes | No | Yes | Yes | No | **No** |
| Misra | 2016 | 27618918 | Low | Low | Low | NA | **Low** | Yes | No | Yes | Yes | No | **No** |
| Ragupathi D | 2016 | 27530999 | Low | Low | Moderate | NA | **Low** | Yes | NA | Yes | No | Yes | **No** |
| [Elumalai S](https://www.ncbi.nlm.nih.gov/pubmed/?term=Elumalai%20S%5BAuthor%5D&cauthor=true&cauthor_uid=27166067) | 2016 | 27166067 | Low | Low | Moderate | NA | **Low** | Yes | NA | Yes | No | No | **Yes** |
| [Al-Emran HM](https://www.ncbi.nlm.nih.gov/pubmed/?term=Al-Emran%20HM%5BAuthor%5D&cauthor=true&cauthor_uid=26933020) | 2016 | 26933020 | Low | Low | Low | Low | **Low** | Yes | Yes | Yes | No | Yes | **No** |
| Thanh D | 2016 | 26974227 | Low | Low | Low | Low | **Low** | Yes | No | Yes | No | Yes | **No** |
| Wong V | 2016 | 27657909 | Low | Low | Low | Low | **Low** | Yes | No | Yes | Yes | Yes | **No** |
| [García-Fernández A](https://www.ncbi.nlm.nih.gov/pubmed/?term=Garc%C3%ADa-Fern%C3%A1ndez%20A%5BAuthor%5D&cauthor=true&cauthor_uid=26121266) | 2015 | 26121266 | Low | Low | Low | Low | **Low** | Yes | No | Yes | No | No | **Yes** |
| [Nüesch-Inderbinen M](https://www.ncbi.nlm.nih.gov/pubmed/?term=N%C3%BCesch-Inderbinen%20M%5BAuthor%5D&cauthor=true&cauthor_uid=25963025) | 2015 | 25963025 | Low | Low | Low | Low | **Low** | Yes | No | Yes | No | No | **Yes** |
| [Akinyemi KO](https://www.ncbi.nlm.nih.gov/pubmed/?term=Akinyemi%20KO%5BAuthor%5D&cauthor=true&cauthor_uid=25999745) | 2015 | 25999745 | Low | Low | Low | Low | **Low** | Yes | No | Yes | No | No | **Yes** |
| [Ceyssens PJ](https://www.ncbi.nlm.nih.gov/pubmed/?term=Ceyssens%20PJ%5BAuthor%5D&cauthor=true&cauthor_uid=25385108) | 2015 | 25385108 | Low | Low | Low | Low | **Low** | Yes | No | Yes | No | No | **Yes** |
| Wong V | 2015 | 25961941 | Low | Low | Low | Low | **Low** | Yes | No | Yes | Yes | Yes | **No** |
| [Chiou CS](https://www.ncbi.nlm.nih.gov/pubmed/?term=Chiou%20CS%5BAuthor%5D&cauthor=true&cauthor_uid=25136011) | 2014 | 25136011 | Low | Low | Low | Low | **Low** | Yes | No | Yes | No | No | **No** |
| Dutta S | 2014 | 25098613 | Low | Low | Low | Low | **Low** | Yes | No | Yes | No | No | **No** |
| [González-López JJ](https://www.ncbi.nlm.nih.gov/pubmed/?term=Gonz%C3%A1lez-L%C3%B3pez%20JJ%5BAuthor%5D&cauthor=true&cauthor_uid=25340972) | 2014 | 25340972 | Low | Low | Low | Low | **Low** | Yes | No | Yes | No | No | **No** |
| [Dahiya S](https://www.ncbi.nlm.nih.gov/pubmed/?term=Dahiya%20S%5BAuthor%5D&cauthor=true&cauthor_uid=25027085) | 2014 | 25027085 | Low | Low | Low | Low | **Low** | Yes | No | Yes | No | No | **No** |
| Saleh FO | 2014 | 24820472 | Low | Low | Low | Low | **Low** | Yes | No | Yes | No | No | **No** |
| [Geetha VK](https://www.ncbi.nlm.nih.gov/pubmed/?term=Geetha%20VK%5BAuthor%5D&cauthor=true&cauthor_uid=24399384) | 2014 | 24399384 | Low | Low | Low | Low | **Low** | Yes | No | Yes | No | No | **No** |
| Lee CJ | 2013 | 23465712 | Low | Low | Low | Low | **Low** | Yes | No | Yes | No | No | **No** |
| [Jain S](https://www.ncbi.nlm.nih.gov/pubmed/?term=Jain%20S%5BAuthor%5D&cauthor=true&cauthor_uid=24240035) | 2013 | 24240035 | Low | Low | Low | Low | **Low** | Yes | No | Yes | Yes | No | **No** |
| [Lunguya O](https://www.ncbi.nlm.nih.gov/pubmed/?term=Lunguya%20O%5BAuthor%5D&cauthor=true&cauthor_uid=23166855) | 2012 | 23166855 | Low | Low | Low | Low | **Low** | Yes | No | Yes | No | No | **No** |
| [Vlieghe ER](https://www.ncbi.nlm.nih.gov/pubmed/?term=Vlieghe%20ER%5BAuthor%5D&cauthor=true&cauthor_uid=23272255) | 2012 | 23272255 | Low | Low | Low | Low | **Low** | Yes | No | Yes | No | No | **No** |
| Emary K | 2012 | 23122884 | Low | Low | Low | Low | **Low** | Yes | No | Yes | Yes | No | **No** |
| [Thamizhmani R](https://www.ncbi.nlm.nih.gov/pubmed/?term=Thamizhmani%20R%5BAuthor%5D&cauthor=true&cauthor_uid=22885270) | 2012 | 22885270 | Low | Low | Low | Low | **Low** | Yes | No | Yes | No | No | **No** |
| [Tatavarthy A](https://www.ncbi.nlm.nih.gov/pubmed/?term=Tatavarthy%20A%5BAuthor%5D&cauthor=true&cauthor_uid=22649021) | 2012 | 22649021 | Low | Low | Low | Low | **Low** | Yes | No | Yes | No | No | **No** |
| [Acharya D](https://www.ncbi.nlm.nih.gov/pubmed/?term=Acharya%20D%5BAuthor%5D&cauthor=true&cauthor_uid=22627312) | 2012 | 22627312 | Low | Low | Low | Low | **Low** | Yes | No | Yes | No | No | **No** |
| Ahmed D | 2012 | 22442289 | Low | Low | Moderate | NA | **Low** | Yes | NA | Yes | No | Yes | **Yes** |
| [Koirala KD](https://www.ncbi.nlm.nih.gov/pubmed/?term=Koirala%20KD%5BAuthor%5D&cauthor=true&cauthor_uid=22371897) | 2012 | 22371897 | Low | Low | Moderate | NA | **Low** | Yes | NA | Yes | No | Yes | **Yes** |
| [Kumarasamy K](https://www.ncbi.nlm.nih.gov/pubmed/?term=Kumarasamy%20K%5BAuthor%5D&cauthor=true&cauthor_uid=22146877) | 2012 | 22146877 | Low | Low | Low | Low | **Low** | Yes | No | Yes | No | No | **No** |
| [Accou-Demartin M](https://www.ncbi.nlm.nih.gov/pubmed/?term=Chau%20TT%5BAuthor%5D&cauthor=true&cauthor_uid=17908946) | 2011 | 21749778 | Low | Low | Low | Low | **Low** | Yes | No | Yes | No | No | **No** |
| [Hassing RJ](https://www.ncbi.nlm.nih.gov/pubmed/?term=Chau%20TT%5BAuthor%5D&cauthor=true&cauthor_uid=17908946) | 2011 | 21227657 | Low | Low | Low | Low | **Low** | Yes | No | Yes | No | No | **No** |
| [Mohanty S](https://www.ncbi.nlm.nih.gov/pubmed/?term=Mohanty%20S%5BAuthor%5D&cauthor=true&cauthor_uid=20828458) | 2010 | 20828458 | Low | Low | Moderate | NA | **Low** | Yes | NA | Yes | No | Yes | **Yes** |
| [Gaborieau V](https://www.ncbi.nlm.nih.gov/pubmed/?term=Gaborieau%20V%5BAuthor%5D&cauthor=true&cauthor_uid=20724089) | 2010 | 20724089 | Low | Low | Moderate | NA | **Low** | Yes | NA | Yes | No | Yes | **Yes** |
| [Nath G](https://www.ncbi.nlm.nih.gov/pubmed/?term=Nath%20G%5BAuthor%5D&cauthor=true&cauthor_uid=20188522) | 2010 | 20188522 | Low | Low | Low | Low | **Low** | Yes | No | Yes | No | No | **No** |
| [Morita M](https://www.ncbi.nlm.nih.gov/pubmed/?term=Morita%20M%5BAuthor%5D&cauthor=true&cauthor_uid=20585124) | 2010 | 20585124 | Low | Low | Moderate | NA | **Low** | Yes | NA | Yes | No | Yes | **Yes** |
| Wu W | 2010 | 20113512 | Low | Low | Low | Low | **Low** | Yes | No | Yes | No | No | **No** |
| [Dimitrov T](https://www.ncbi.nlm.nih.gov/pubmed/?term=Dimitrov%20T%5BAuthor%5D&cauthor=true&cauthor_uid=19889623) | 2010 | 19889623 | Low | Low | Low | Low | **Low** | Yes | No | Yes | No | No | **No** |
| [Pfeifer Y](https://www.ncbi.nlm.nih.gov/pubmed/?term=Pfeifer%20Y%5BAuthor%5D&cauthor=true&cauthor_uid=19788837) | 2009 | 19788837 | Low | Low | Moderate | NA | **Low** | Yes | NA | Yes | No | Yes | **Yes** |
| [Yanagi D](https://www.ncbi.nlm.nih.gov/pubmed/?term=Yanagi%20D%5BAuthor%5D&cauthor=true&cauthor_uid=19631095) | 2009 | 19631095 | Low | Low | Low | Low | **Low** | Yes | No | Yes | No | No | **No** |
| [Yoon HJ](https://www.ncbi.nlm.nih.gov/pubmed/?term=Shanahan%20PM%5BAuthor%5D&cauthor=true&cauthor_uid=10722124) | 2009 | 19259362 | Low | Low | Moderate | NA | **Low** | Yes | NA | Yes | No | Yes | **Yes** |
| [Dimitrov T](https://www.ncbi.nlm.nih.gov/pubmed/?term=Shanahan%20PM%5BAuthor%5D&cauthor=true&cauthor_uid=10722124) | 2009 | 18971360 | Low | Low | Moderate | NA | **Low** | Yes | NA | Yes | No | Yes | **Yes** |
| [Capoor MR](https://www.ncbi.nlm.nih.gov/pubmed/?term=Shanahan%20PM%5BAuthor%5D&cauthor=true&cauthor_uid=10722124) | 2009 | 18687156 | Low | Low | Low | Low | **Low** | Yes | No | Yes | No | No | **No** |
| [Dashti AA](https://www.ncbi.nlm.nih.gov/pubmed/?term=Shanahan%20PM%5BAuthor%5D&cauthor=true&cauthor_uid=10722124) | 2008 | 18606582 | Low | Low | Low | Low | **Low** | Yes | No | Yes | No | No | **No** |
| [Rotimi VO](https://www.ncbi.nlm.nih.gov/pubmed/?term=Rotimi%20VO%5BAuthor%5D&cauthor=true&cauthor_uid=18566147) | 2008 | 18566147 | Low | Low | Moderate | NA | **Low** | Yes | NA | Yes | No | Yes | **Yes** |
| [Dutta S](https://www.ncbi.nlm.nih.gov/pubmed/?term=Dutta%20S%5BAuthor%5D&cauthor=true&cauthor_uid=18280709) | 2008 | 18280709 | Low | Low | Moderate | NA | **Low** | Yes | NA | Yes | No | Yes | **Yes** |
| Al-Sanouri | 2008 | 19741292 | Low | Low | Low | Low | **Low** | Yes | No | Yes | No | No | **No** |
| [Chau TT](https://www.ncbi.nlm.nih.gov/pubmed/?term=Chau%20TT%5BAuthor%5D&cauthor=true&cauthor_uid=17908946) | 2007 | 17908946 | Low | Low | Low | Low | **Low** | Yes | No | Yes | No | No | **No** |
| Chau TT | 2007 | 17908946 | Low | Low | Low | Low | **Low** | Yes | No | Yes | No | No | **No** |
| Chau TT | 2007 | 17908946 | Low | Low | Low | Low | **Low** | Yes | No | Yes | Yes | No | **No** |
| [Capoor MR](https://www.ncbi.nlm.nih.gov/pubmed/?term=Capoor%20MR%5BAuthor%5D&cauthor=true&cauthor_uid=17873998) | 2007 | 17873998 | Low | Low | Low | Low | **Low** | Yes | No | Yes | No | No | **No** |
| [Tamang MD](https://www.ncbi.nlm.nih.gov/pubmed/?term=Tamang%20MD%5BAuthor%5D&cauthor=true&cauthor_uid=17629465) | 2007 | 17629465 | Low | Low | Low | Low | **Low** | Yes | No | Yes | No | No | **No** |
| [Gaind R](https://www.ncbi.nlm.nih.gov/pubmed/?term=Gaind%20R%5BAuthor%5D&cauthor=true&cauthor_uid=17071955) | 2006 | 17071955 | Low | Low | Low | Low | **Low** | Yes | No | Yes | No | No | **No** |
| [Shirakawa T](https://www.ncbi.nlm.nih.gov/pubmed/?term=Shirakawa%20T%5BAuthor%5D&cauthor=true&cauthor_uid=16466897) | 2006 | 16466897 | Low | Low | Low | Low | **Low** | Yes | No | Yes | No | No | **No** |
| [Lee K](https://www.ncbi.nlm.nih.gov/pubmed/?term=Lee%20K%5BAuthor%5D&cauthor=true&cauthor_uid=15504831) | 2004 | 15504831 | Low | Low | Low | Low | **Low** | Yes | No | Yes | No | No | **No** |
| [Renuka K](https://www.ncbi.nlm.nih.gov/pubmed/?term=Renuka%20K%5BAuthor%5D&cauthor=true&cauthor_uid=15256030) | 2004 | 15256030 | Low | Low | Low | Low | **Low** | Yes | No | Yes | No | No | **No** |
| [Mills-Robertson F](https://www.ncbi.nlm.nih.gov/pubmed/?term=Mills-Robertson%20F%5BAuthor%5D&cauthor=true&cauthor_uid=12399042) | 2002 | 12399042 | Low | Low | Low | Low | **Low** | Yes | No | Yes | No | No | **No** |
| [Shanahan PM](https://www.ncbi.nlm.nih.gov/pubmed/?term=Shanahan%20PM%5BAuthor%5D&cauthor=true&cauthor_uid=10722124) | 2000 | 10722124 | Low | Low | Low | Low | **Low** | Yes | No | Yes | No | No | **No** |
| Shanahan PM | 2000 | 10722124 | Low | Low | Low | Low | **Low** | Yes | No | Yes | No | No | **No** |
| Shanahan PM | 2000 | 10722124 | Low | Low | Low | Low | **Low** | Yes | No | Yes | No | No | **No** |
| Shanahan PM | 2000 | 10722124 | Low | Low | Low | Low | **Low** | Yes | No | Yes | No | No | **No** |
| Shanahan PM | 2000 | 10722124 | Low | Low | Low | Low | **Low** | Yes | No | Yes | No | No | **No** |
| Shanahan PM | 1998 | 9620383 | Low | Low | Low | Low | **Low** | Yes | No | Yes | No | No | **No** |
| [Wain J](https://www.ncbi.nlm.nih.gov/pubmed/?term=Wain%20J%5BAuthor%5D&cauthor=true&cauthor_uid=9431387) | 1997 | 9431387 | Low | Low | Low | Low | **Low** | Yes | No | Yes | No | No | **No** |
| [Panigrahi D](https://www.ncbi.nlm.nih.gov/pubmed/?term=Panigrahi%20D%5BAuthor%5D&cauthor=true&cauthor_uid=8765450) | 1996 | 8765450 | Low | Low | Low | Low | **Low** | Yes | No | Yes | No | No | **No** |

**Quality In Prognosis Studies tool (QUIPS) Domains : (Hayden et al, 2013)**

| *Domain 1:*  **Study population** | The study sample sufficiently represents the population of interest to minimise the potential risk of bias to the results. |
| --- | --- |
| *Domain 2:*  **Measurement of outcomes** | The outcomes of interest measured adequately and similarly for all isolates in the study to minimise potential risk of bias to the results. |
| ***Domain 3:***  **Study Attrition** | The available study data sufficiently represent the entire study sample |
| *Domain 4:*  **Exposure measurement** | *Not applicable to this systematic review* |
| *Domain 5:*  **Statistical data, analysis and reporting** | Reliable data, appropriate statistical analysis for study design, and all outcome measures are reported |

**Joanna Briggs Institute (JBI) tool (The Joanna Briggs Institute - Reviewer’s manual) and modifications from Tadesse *et al, 2018***

| *Quality item 1:* | Was the sampling/target population described? |
| --- | --- |
| *Quality item 2:* | Was sampling prospective? |
| *Quality item 3:* | Was microbiological culture and adequate quality control of disc diffusion/MIC methods applied? |
| *Quality item 4:* | Was the sample size >200 for Objective 1 and > 100 for phenotype 2 |
| *Quality item 5:* | Performance standards adequately and appropriately described for objective 1 and whether whole genome sequencing was used for objective 2 |

The first tool aids in reporting whether each study has a low, moderate or high risk of bias while the second tool reports whether there is a risk of bias or not as a simple binary outcome.
