# Supplementary material for: A systematic review of antimicrobial resistance in Salmonella enterica serovar Typhi, the etiological agent of typhoid
Source: PLoS Negl Trop Dis. 2018 Oct 11;12(10):e0006779. doi: 10.1371/journal.pntd.0006779 (PMC6198998; doi:10.1371/journal.pntd.0006779)

**Supplementary Figure 1: Confidence intervals of the year stratified summaries in each temporal period.**

Those including 0 do not contribute significantly to the overall trends in Figure 2 and did not change the overall trends on sensitivity analyses


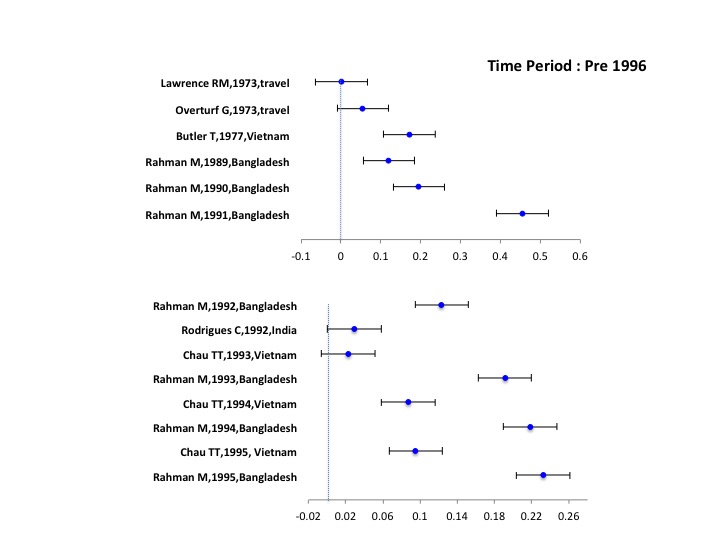


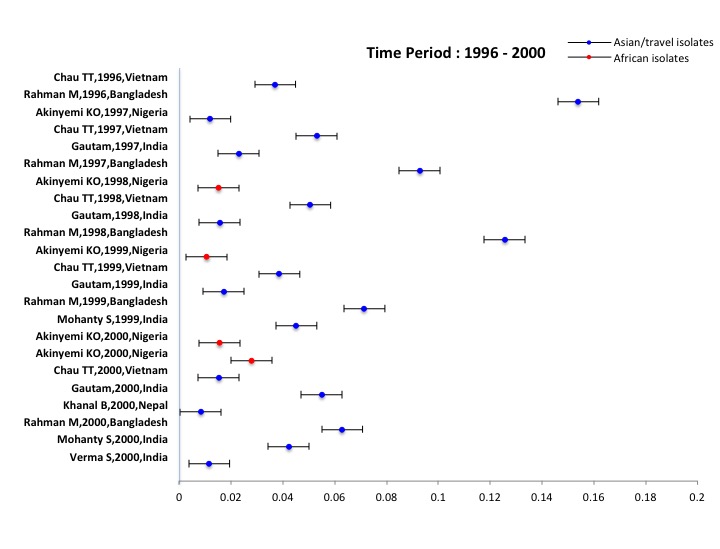


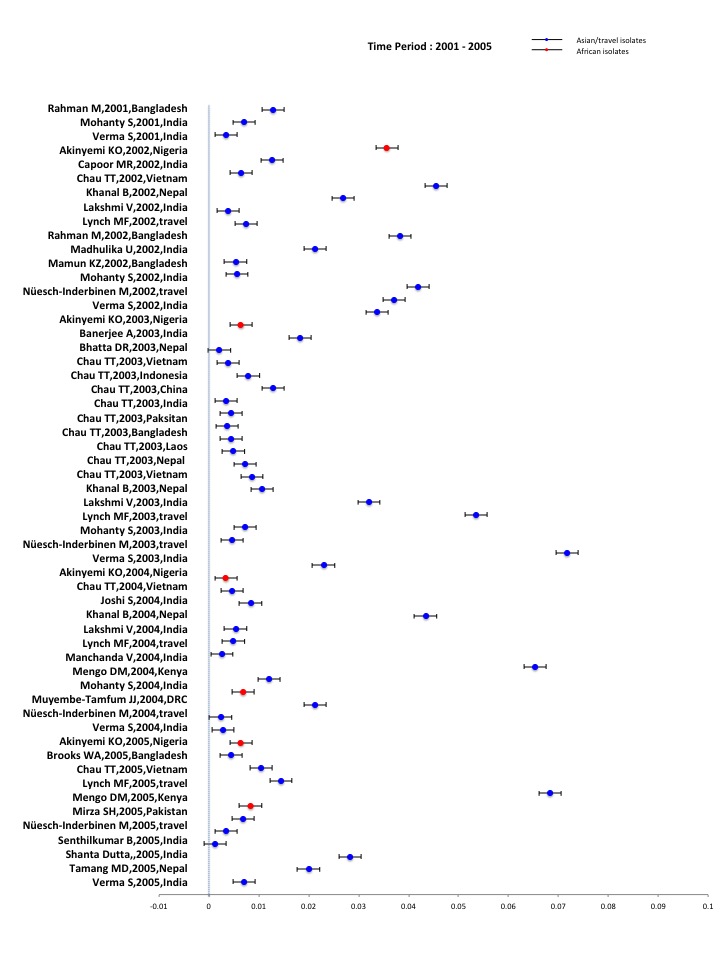


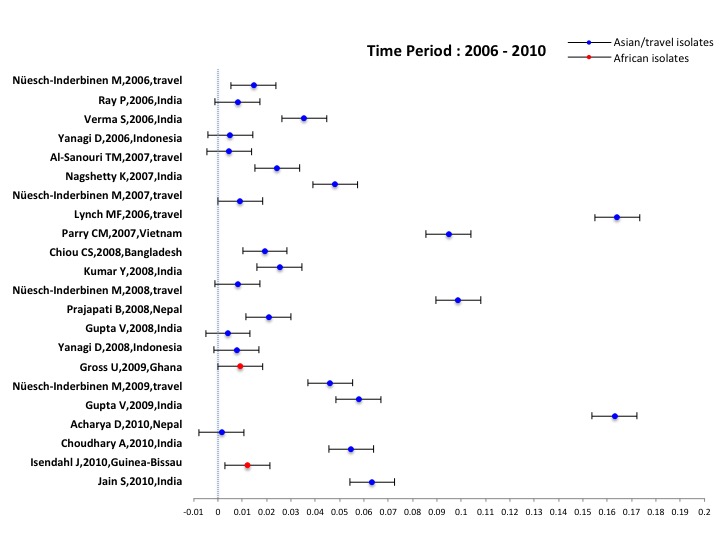


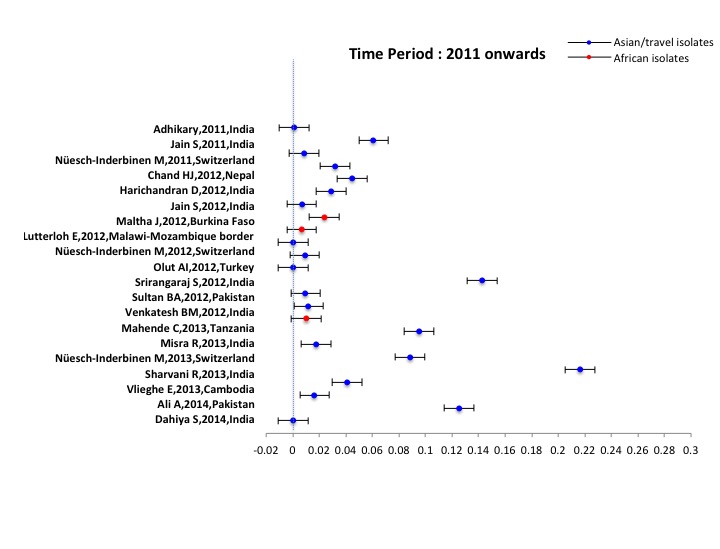

Supplement: S1 Fig — Those including 0 do not contribute significantly to the overall trends in Fig 2 and did not change the overall trends on sensitivity analyses. (DOCX) [file pntd.0006779.s006.docx]
