# Supplementary figures and images for: A systematic review of antimicrobial resistance in Salmonella enterica serovar Typhi, the etiological agent of typhoid
Source: PLoS Negl Trop Dis. 2018 Oct 11;12(10):e0006779. doi: 10.1371/journal.pntd.0006779 (PMC6198998; doi:10.1371/journal.pntd.0006779)

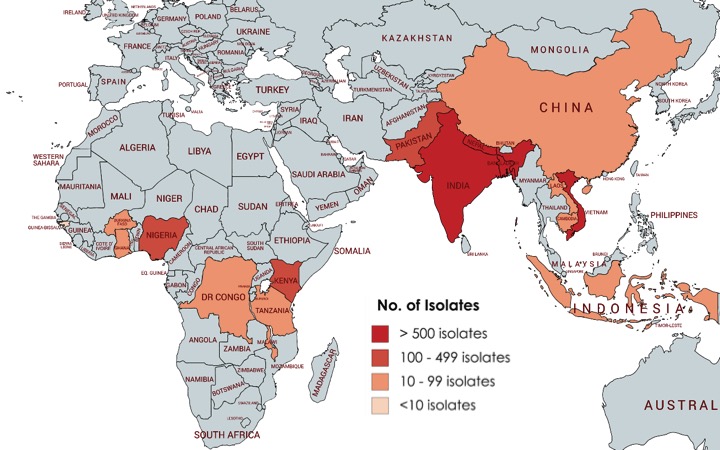

Supplement: S2 Fig — (JPG) [file pntd.0006779.s007.jpg]
